# Maternal obesity during lactation may protect offspring from high fat diet-induced metabolic dysfunction

**DOI:** 10.1038/s41387-018-0027-z

**Published:** 2018-04-25

**Authors:** Jenifer Monks, David J. Orlicky, Adrianne L. Stefanski, Andrew E. Libby, Elise S. Bales, Michael C. Rudolph, Ginger C. Johnson, Vanessa D. Sherk, Matthew R. Jackman, Kayla Williamson, Nichole E. Carlson, Paul S. MacLean, James L. McManaman

**Affiliations:** 1 Division of Reproductive Sciences, Department of Obstetrics & Gynecology, School of Medicine, University of Colorado Denver Anschutz Medical Campus, Aurora, CO 80045 USA; 2 Pathology Department, School of Medicine, University of Colorado Denver Anschutz Medical Campus, Aurora, CO 80045 USA; 3 Department of Medicine, University of Colorado School of Medicine, Aurora, CO USA; 4 Division of Endocrinology, Metabolism, & Diabetes, Department of Medicine, School of Medicine, University of Colorado Denver Anschutz Medical Campus, Aurora, CO 80045 USA; 5 Department of Biostatistics and Informatics, University of Colorado Anschutz Medical Campus, Aurora, CO USA

## Abstract

**Background/Objectives:**

The current obesity epidemic has spurred exploration of the developmental origin of adult heath and disease. A mother’s dietary choices and health can affect both the early wellbeing and lifelong disease-risk of the offspring.

**Subjects/Methods:**

To determine if changes in the mother’s diet and adiposity have long-term effects on the baby’s metabolism, *independently* from a prenatal insult, we utilized a mouse model of diet-induced-obesity and cross-fostering. All pups were born to lean dams fed a low fat diet but were fostered onto lean or obese dams fed a high fat diet. This study design allowed us to discern the effects of a poor diet from those of mother’s adiposity and metabolism. The weaned offspring were placed on a high fat diet to test their metabolic function.

**Results:**

In this feeding challenge, all male (but not female) offspring developed metabolic dysfunction. We saw increased weight gain in the pups nursed on an obesity-resistant dam fed a high fat diet, and increased pathogenesis including liver steatosis and adipose tissue inflammation, when compared to pups nursed on either obesity-prone dams on a high fat diet or lean dams on a low fat diet.

**Conclusion:**

Exposure to maternal over-nutrition, through the milk, is sufficient to shape offspring health outcomes in a sex- and organ-specific manner, and milk from a mother who is obesity-prone may partially protect the offspring from the insult of a poor diet.

## Introduction

The nursing period is an important developmental step between the controlled and protected, prenatal environment and exposed independence post-weaning. Essential information is passed from mother to offspring, in the milk, influencing physiological systems, to ready the neonate for independent survival^1^. Immune sensitization or tolerance and the infant microbiome are influenced by components of mother’s milk^2,3^. Additionally, nutritional composition of the milk is thought to influence eating habits, nutrient utilization, and fat deposition^4–6^.

Experiments have shown that prenatal insults transmitted through the placenta, have a definitive effect on metabolic function into adulthood^7,8^. Dissection of the information passed through the milk requires cross-fostering, to avoid these prenatal effects. However, controlled, cross-fostering experiments in humans are a challenge due to ethical considerations^9^. We have chosen a mouse model, which allows precise control of genetics, diet, and environment, in order to tease out subtle effects of changes in the mother’s metabolism, communicated through the milk to her offspring^10^. In previous studies^11^, we demonstrated that maternal obesity/adiposity, above and beyond the consequences of a high-fat diet, influences neonatal metabolism. Here we use the same experimental paradigm to generate diet-induced obese (DIO) dams, but to isolate the immediate post-partum period, we studied only pups born to low fat-fed, lean mothers. These pups were cross-fostered onto lean and obese mothers, fed low fat or high fat diets, in order to differentiate the effects of a poor diet from mother’s adiposity. Immediately upon weaning, the pups were challenged with a high-fat diet to measure phenotypic plasticity and to assess their susceptibility to metabolic dysfunction.

## Methods

### Ethical approval

All animals were conventionally housed in the University of Colorado Denver Anschutz Medical Campus, AAALAC-accredited, Center for Comparative Medicine and all procedures were approved by the Institutional Animal Care and Use Committee. Mice had ad libitum access to food, in micro-isolator cages with automated air and water, on a 10:14 h dark:light cycle, maintained at 72 °F. All animals were provided with cotton nesting material enrichment and breeders and experimental females were additionally provided with shredded paper to construct an enclosed nest.

### Diet-induced obesity with C57Bl/6J dams

Female C57Bl/6 J mice, which have heterogeneous weight gain and metabolic responses to high fat diet feeding^12^ were used to generate obesity prone and obesity resistant dams as previously described^11^. Briefly, 7 week-old females were purchased from Jackson Laboratories. At 8 weeks old, mice were assigned to long-term HFD or LFD (Research Diets 60% kcal fat and 10% kcal fat, lard-based, D12492i and D12450Bi). After 6 weeks, HFD-fed animals were sorted based on rate of weight gain. The lowest tertile was designated obesity resistant (OR), the highest tertile was designated obesity prone (OP), and the middle tertile was humanely euthanized^11^. In this previous study^11^ we saw significant differences in measured outcomes with 4–6 dams/litters per group. Thus we had, in each group 5–6 dams. These diet-conditioned mice were placed with chow-fed, non-obese, stud males (C57Bl/6 J) for one estrous cycle (4 nights) to synchronize births. LFD-fed lean (LN) mice were mated synchronously to generate all donor pups. Litters born to LFD-fed LN dams on a single day were combined and randomly assigned to foster dams. Litters were standardized to 5 pups (either 2M3F or 3M2F), cross-fostered on day 3, and dams and litters were weighed daily. All weanling pups were singly housed, placed immediately onto the 60% kcal fat challenge diet, and were weighed weekly. Litters from both non-parous and primiparous dams were used, so birthdates varied between offspring. Two animals (LN:LN) were recommended for early sacrifice by veterinarians, due to skin lesions and these animals were not included in the final data analysis. Three other males (one LN:LN and two LN:OR) were found, upon necropsy, to have enlarged spleens and/or kidneys. These animals were not excluded. Vaginal cytology was used to determine estrus; females which were not cycling were excluded. Glucose tolerance tests, body fat measurement by QMR, and calorimetry were performed on a separate cohort of males after 10 weeks on HFD. Animals were sacrificed and tissue analyses were performed after 10 or 20 weeks.

For our primary outcome of weight with 5 mice per group we have 80% power to detect a 4.65 g difference in the means of two groups using a two-sample t-test with an alpha = 0.025 (to adjust for multiple comparisons) based on preliminary data with an SD = 2. This is a large effect size.

### Milk analysis

A separate group of DIO dams were generated (as above) for milking. These dams had identical growth trajectories and body weights to those nursing offspring above. (data not shown) Litters were standardized to 5 pups on day 3 and mice were milked on day 10. Pups were removed from dams for 3 h to allow milk accumulation in the mammary glands. Milk removal was as described (^13^, Method 2) except that xylazine was used^14^. Litters were humanely euthanized and milked dams were allowed 7 days to recover and regress and then re-bred for a 2^nd^ parity.

Aliquots of whole milk were frozen at −80 °C. Additionally, 100 uL of whole milk was mixed with 100 uL sterile PBS with protease inhibitors (Sigma) and centrifuged at 14,000 × g for 20 minutes, to separate the cream and milk proteins. The cleared whey fraction was collected and frozen at −80 °C. Leptin and insulin were measured in the whey fraction using commercially available kits. (Alpco Diagnostics, Windam, NJ) Aliquots of whole milk were used to measure protein, lactose and TAG content. The fatty acid make-up of the neutral lipid fraction was analyzed by Gas Chromatography Mass Spectrometry as previously described^15,16^.

### Histology and Immunohistochemistry

Samples from freshly excised tissues were fixed for 24–36 h in 4% paraformaldehyde, subsequently embedded in paraffin, and sectioned as described previously^18^. Sectioning and H&E staining of tissue samples were performed by the Pathology Core at the University of Colorado Denver Anschutz Medical Campus. Liver damage was scored as previously described^17,18^. Briefly, the extent of steatosis, the presence of inflammatory cells and foci, features of hepatocyte injury, and markers of tissue response, were determined by cell morphology and staining by a trained histopathologist; an entire section of liver from 10–14 mice/group was examined and the extent of each was scored by the histopathologist (blinded). Similarly for characterization of brown adipose tissue morphology. Histologic images were captured on an Olympus BX51 microscope equipped with a 4MP Macrofire digital camera (Optronics) using the PictureFrame Application 2.3 (Optronics). H&E-stained adipose tissue depots were scored (blindly) for evidence of pathology/inflammation, using 5–12 fields covering one section from each animal.

Immunostaining, imaging and image analysis for crown structures was as previously described^14^. Stains used: guinea pig antibody to Plin1 (Fitzgerald, 1:200, 20R-PP004), Wheat germ agglutinin (Alexa 555 conjugate, Invitrogen), and DAPI (Sigma, D9542). Imaging was performed on a Marianas Spinning Disc Confocal microscope (Intelligent Imaging Innovations, Inc., Denver, CO). Analysis was performed using SlideBook v. 6.0 (Intelligent Imaging Innovations, Inc.), on 5 images from 12 animals/group.

### Calorimetry

Measures of energy balance and fuel utilization were performed as described in previous reports^19,20^. For measurements of energy balance, mice were acclimatized to the Colorado Nutrition Obesity Research Center animal satellite facility for several days before being placed in the eight-chamber metabolic monitoring system (Oxymax CLAMS- 8 M; Columbus Instruments). All mice remained in the chambers for 7 days, with at least 2 days of acclimatization to the new environment and at least 3 days for measurements of energy intake and expenditure. Twenty four hour urine was collected in the chambers, and urinary urea nitrogen, and creatinine were determined (ThermoElectron, Melbourne, Australia). Metabolic rate (MR) was measured every 16 min and calculated with the Weir equation (MR = 3.941 × vO2 + 1.106 × vCO2 − 2.17 × N), where *N* = urinary nitrogen^21^. MR averaged over the day was then extrapolated throughout the 24 h testing period to acquire estimates of total energy expenditure (TEE) described previously^19^. Body composition was determined in dams, litters, and adult offspring by quantitative magnetic resonance (qMR; Echo MRI Whole Body Composition Analyzer; Echo Medical Systems, Houston, TX, USA).

### RNA isolation and gene expression

For adipose tissue RNA isolation, 50–75 mg of tissue was homogenized into Qiazol reagent (Qiagen) using bead homogenization. The homogenate was transferred to phase lock gel heavy tubes (Quanta BioSciences) and mixed with 400 uL chloroform by vigorous shaking. The samples were then centrifuged at 16,000 xg for 20 min at 4 °C. The top aqueous layer above the gel was removed and mixed 1:1 with Buffer RLT Plus (Qiagen). Samples were then mixed with 1 mL 70% ethanol and subjected to cleanup using the RNeasy Plus mini kit (Qiagen) per kit instructions. Genomic DNA was removed by on-column DNase digestion. 1 ug of RNA was used for cDNA synthesis using the iScript cDNA synthesis kit (Bio-Rad). qRT-PCR was run on a CFX96 instrument (Bio-Rad) using 2 × SYBR Green qPCR Mastermix (BioTool). Gene expression was normalized to 18 S rRNA. Primers sequences were as follows:

[TNFα-F: TCTCAGCCTCTTCTCATTCCTGCT, TNFα-R: AGAACTGATGAGAGGGAGGCCATT, F4/80-F: TCAAATGGATCCAGAAGGCTCCCA, F4/80-R: TGCACTGCTTGGCATTGCTGTATC, Timp1-F: CTCAAAGACCTATAGTGCTGGC, Timp1-R: CAAAGTGACGGCTCTGGTAG, 18S-F: CGGCTTAATTTGACTCAACAC, 18S-R: ATCAATCTGTCAATCCTGTCC.]

### Statistical analysis

Statistical analyses and graphing were performed using using R studio version 3.4.0 (2017-04-21) (R Core Team (2017) or using GraphPad Prism v. 7.0a. Unless otherwise indicated, graphs represent the mean and the standard error of the mean. ANOVA was used to assess whether there were significant mean differences between any of the three groups (LN:LN, LN:OR, LN:OP). When the ANOVA was significant, Pairwise Student’s t-tests and Bonferonni adjustment were used to make comparisons between LN:OR and LN:OP (e.g., on dams differing in body weight, and the offspring they nursed) and LN:OR vs LN:LN (dams differing in diet and the offspring they nursed). *P* < 0.05 was used for statistical significance in the ANOVA and a *P* < 0.025 for the pairwise comparisons. Only two comparisons were adjusted for because they are the groups that were a priori hypothesized to be of interest. A separate analysis was performed for each outcome. Additional ANOVA analyses were performed stratified on tests made between males and females, and comparisons between 10 wk and 20 wk HFD animals within a single group were conducted. Because the differences between offspring groups could be due to variability of weight gain rather than maternal parameters, Linear Mixed Models with a random intercept were used to assess body weight differences, adjusting for baseline weight, post-puberty, weeks 7–20 of HFD, between group, over time, stratifying on gender. Kruskal-Wallis tests with Dunn’s multiple comparison testing were also used to compare liver injury scores between groups.

## Results

**Fig. 1:**
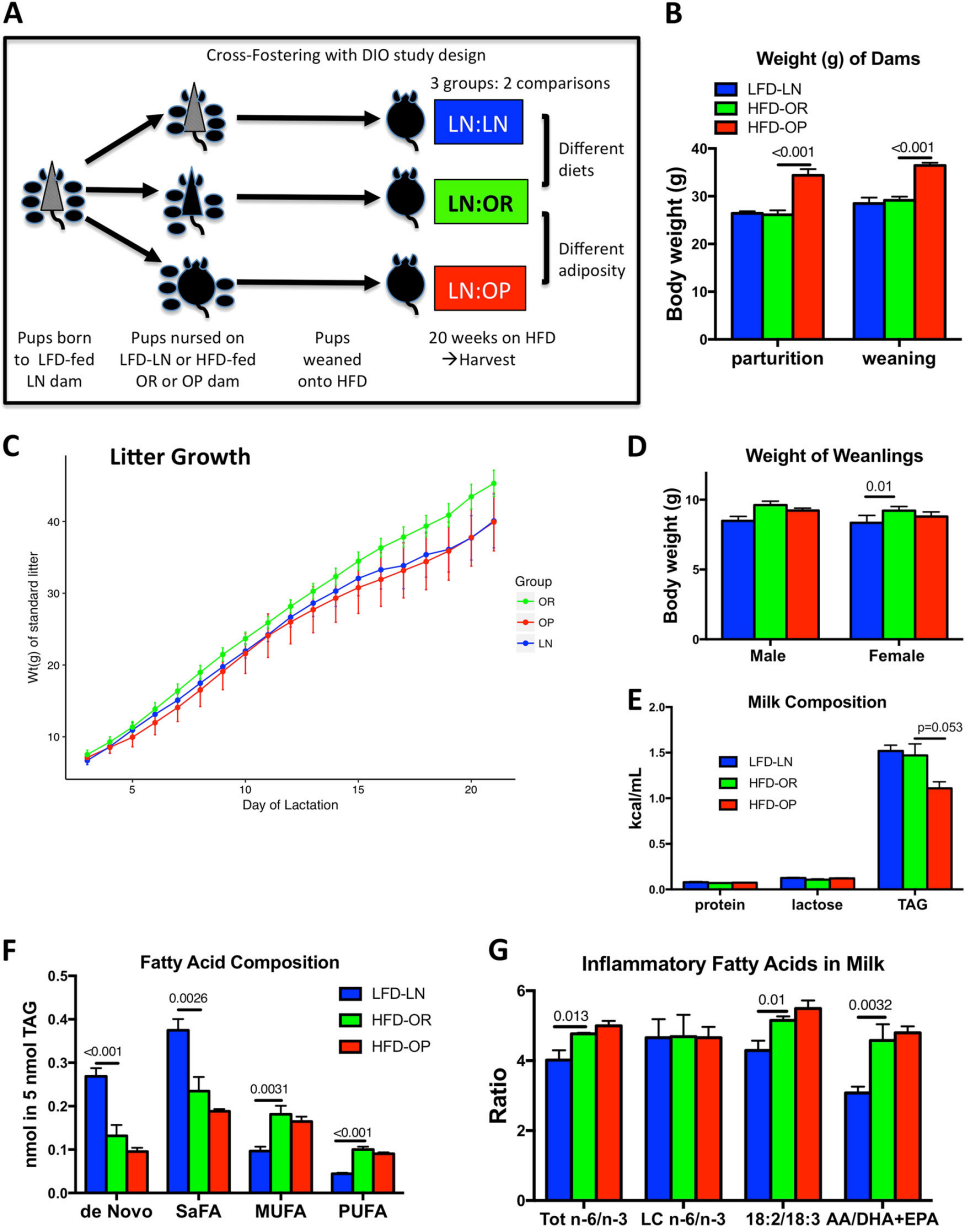
Study design and generation of DIO dams. **a** Diagram of study design: Pups born to LFD-fed, lean dams (LFD-LN) were cross-fostered to nurse upon HFD-fed, obesity resistant (HFD-OR) or obesity prone (HFD-OP) dams. Upon weaning, all cross-fostered offspring were placed onto HFD for 20 weeks. **b** Body weight of dams at parturition and at weaning: LFD-LN (blue), HFD-OR (green), or HFD-OP (red) dams. **c** Weight of standardized litters (g), shown for the entire course of lactation, of pups nursed on LFD-LN (blue), HFD-OR (green), or HFD-OP (red) dams. Graphs show the mean ± s.e.m. for 4–5 dams/group. **d** Weight (g) at weaning of cross-fostered male and female pups. **e** Milk composition analysis from LFD-LN (blue), HFD-OR (green), or HFD-OP (red) dams, showing total protein, lactose and total triglyceride, expressed as kcal/mL of milk. **f** Fatty Acid composition analysis of the neutral lipid fraction of milk. Data are expressed in nmoles of fatty acid per nmole of milk triglyceride. (De novo = 8:0, 10:0, 12:0, 14:0, SaFA = 8:0, 10:0, 12:0, 14:0, 16:0, 18:0, 20:0, MUFA = 16:1, 18:1, 20:1, PUFA = all polyunsaturated of both n-6 and n-3 series: 18:2, 18:3, 20:2, 20:3, 20:4, 20:5, 22:2, 22:3, 22:4, 22:5, 22:6.) **g** Ratios of bioactive fatty acids from the neutral lipid fraction of milk. The total n-6/n-3 fatty acid ratio is the quantitative sum of n-6 (18:2, 18:3, 20:2, 20:3, 20:4, 22:2, 22:4, and 22:5) divided by the sum of n-3 (18:3, 20:3, 20:4, 20:5, 22:3, 22:5, and 22:6); the AA/DHA + EPA ratio is the absolute amount of 20:4 n-6 divided by the sum of 22:6 n-3 + 20:5 n-3. Graphs show the mean ± s.e.m. for 4–5 milk samples/group

C57Bl/6 mice placed on a high-fat diet are a well-established, inbred mouse model of diet-induced obesity^12,22^. When 8 week-old female mice are given 60% of their caloric intake from lard, they gain weight at a variable rate and segregate into obesity resistant and obesity prone groups^11,23^. We exploited this model to generate genetically identical, high fat-fed, lean and obese animals for directly testing the effects of the mother’s adiposity on the outcome of offspring she nurses. Low fat-fed mice were used to generate all pups, which were then cross-fostered onto three different groups of dams: low fat-fed lean (LFD-LN), high fat-fed obesity-resistant (HFD-OR), and high fat-fed obesity-prone (HFD-OP). (Fig. 1a) This design allowed us to make two, single-variant comparisons:

1. pups nursed on lean dams with low-fat or high fat diet,
2. pups nursed on high fat-fed dams that differed in their body fat content and propensity for weight gain.

Importantly, the pups we studied did not have a prenatal insult. All of the dams on a high fat diet showed increased fasting blood glucose levels by 6 weeks, the point at which breeding began. (mean (SEM), LFD-LN: 86.8(±2.5), HFD-OR: 96.6(±1.8), HFD-OP: 100.7(±2.7), *p* = 0.0004) mg/dL. There was a significant difference between dam weights in the OP and OR groups at both parturition and weaning (*p* < 0.001 and *p* < 0.001; Fig. 1b, Suppl.Table 1) as expected. The body fat percentage at conception of HFD-OP dams (21 ± 2%) was significantly greater than HFD-OR (16 ± 4%) dams. The dams gained approximately 2 g of body weight during the nursing period, from parturition to weaning and this was not different between the groups. (Fig. 1b) There was a significant overall difference between groups for weight (in grams) of standard litter over days of lactation (*p* < 0.001; Fig. 1c, Suppl.Table 2). There was a −0.277 g smaller increase per day in weight for the LN group than the OR group (*p* < 0.001). There was a −0.279 g smaller increase per day in weight for the OP group compared to the OR group (*p* < 0.001). For every one day increase there was 1.853 g increase in weight of the standard litter for the LN group. For every one day increase there was 1.851 g increase in weight of the standard litter for the OP group. (Fig. 1c, Suppl. Table 2). There was a significant difference in the average body weight of female weanlings in the LN group compared to the OR group (*p* = 0.01, Fig. 1d, Suppl. Table 3) Gross analysis of milk from a separate set of dams, collected on day 10, showed similar levels of protein, and lactose, however HFD-OP dams produced milk with 24% less triglyceride (Fig. 1e), as previously described^11^. There was a significant difference in milk in fatty acid composition between the LN:LN and LN:OR groups for de Novo (*p* < 0.001; Table 4), SaFA (*p* = 0.0026; Suppl. Table 4), MUFA (*p* = 0.0031; Table 4), and PUFA (*p* < 0.001; Fig. 1f, Suppl. Table 4). There were also significant differences in inflammatory fatty acids in milk between the LN:LN and LN:OR groups Total n-6/n-3 (*p* = 0.013; Fig. 1g, Suppl. Table 4), 18:2/18:3 (*p* = 0.01; Table 4), and AA/DHA + EPA (*p* = 0.0032; Fig. 1g, Suppl. Table 4), as expected^24^. In the HFD-fed dams, increased fatty acids from the diet were utilized for milk fat synthesis and were measured as an increase in stearic acid (18:0), oleic acid (18:1 n-9), and linoleic acid (18:2 n-6) (Table S1), which increased the total levels of MUFA, and PUFA, and increased the n-6/n-3, and 18:2/18:3 ratios. (Fig. 1f, g) Despite the differences in fasting blood glucose of the dams prior to breeding, no difference was seen in the levels of insulin measured in their milks (mean: 2.5 ng/mL, range: 0.5–8 ng/mL). Leptin levels were measured in the whey fraction of the milk at lactation day 10, and were twice as high (*P* < 0.05) in the HFD-fed dams (HFD-OR: 21.7 ± 3.9; HFD-OP: 20.5 ± 4.5 ng/mL) than in the LFD-fed dams (11.6 ± 5.9).

**Fig. 2:**
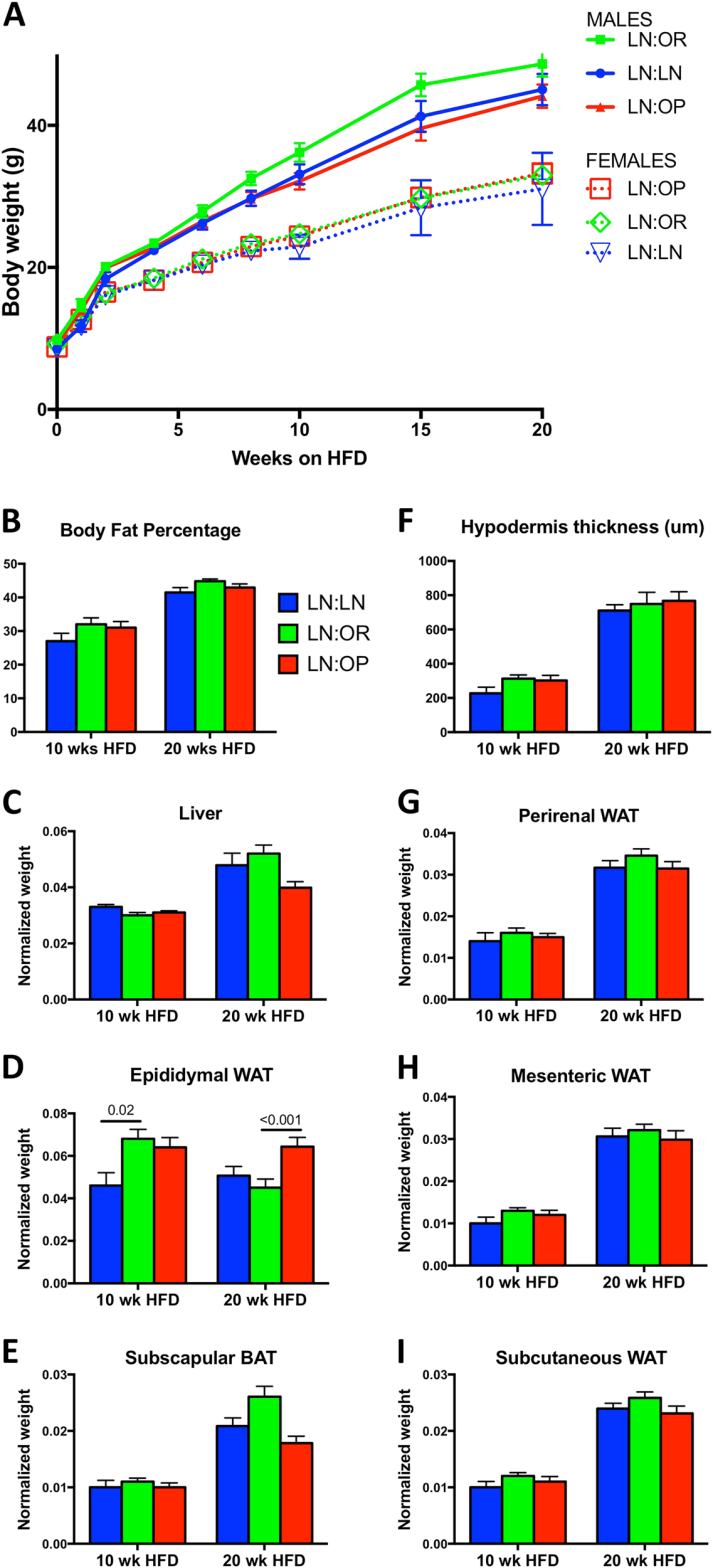
Diet challenge of cross-fostered offspring. **a** Growth of cross-fostered male and female offspring during the HFD challenge. Shown are body weights of LN:LN (blue, *n* = 10 males, 6 females), LN:OR (green, *n* = 11 males, 10 females), or LN:OP (red, *n* = 12 males, 8 females) mice. . **b** Body fat percentage as measured by QMR of LN:LN (blue), LN:OR (green), or LN:OP (red) mice, at 10 or 20 weeks HFD. Shown are the normalized tissue weights (except where noted) of **c** Liver, **d** Epididymal WAT, **e** Subscapular BAT, **f** Hypodermis thickness (microns), **g** Perirenal WAT, **h** Mesenteric WAT, and **i** Subcutaneous WAT

Post-weaning, all of the pups were singly-housed and placed on a HFD. Rapid catch-up growth was displayed by the LN:LN male pups; within 3 weeks on HFD, their weights were comparable to the LN:OR group (data not shown). Body weights of the weanlings began to diverge by 8 weeks. (Fig. 2a) There was a significant difference between the post-pubertal weight gain in males and females over time on HFD (20 weeks) after adjusting for baseline (*p* < 0.001; Suppl. Table 5). There was also a significant difference between groups over time after adjusting for baseline (*p* < 0.001; Suppl. Table 5). Males had an additional 0.492 g increase in weight per week compared to females. The LN:LN group had an additional 0.171 g increase in weight per week compared to the LN:OR group. The LN:OP group had a 0.218 g smaller increase in weight per week compared to the LN:OR group.

Measured food consumption was not significantly different between groups, nor between males and females. (Suppl. Fig. S2) However, weight gain during the post-pubertal period was 20–30% higher for LN:OR males during this period, compared to the other two groups of males (Fig. 2a & S1). Female offspring, despite consuming the same milk, being the same weight at weaning, being singly-housed, being challenged with the same HFD, and consuming the same amount of food, gained weight more slowly (Fig. 2a & S1) than their male siblings.

Body weights of the weanlings were measured for 20 weeks of HFD-feeding, from 5–6 litters/group. The rate of weight gain of the males began to plateau as they reached 50 g, occurring in 7/13 of the LN:LN group, 9/14 of the LN:OR group, and 5/14 of the LN:OP group. (Fig. S1) These differences in development of pathology are consistent with poor nutrition during organ development altering the risk of disease^25,26^. Although not overtly sick, this reduction indicates a change in their overall health status. The animals were sacrificed at this time for tissue analysis.

A separate cohort of male weanlings (7–8 males from 3 dams/group) were subjected to glucose tolerance testing and calorimetry after 10 weeks of HFD to assess differences in metabolism at a point when the rate of weight gain was significantly different, and before any overt signs of illness or inflammation. At this point, all males were glucose-intolerant compared to chow-fed, age-matched controls that were not cross-fostered, but showed no differences between the groups. (data not shown) The respiratory quotient of all groups was 0.80 (0.79 light RQ, 0.81 dark RQ), indicating significant fat-burning on the HFD. The total activity between groups was not different, however the non-ambulatory activity of the LN:LN group was slightly increased over that of the LN:OR group. (data not shown) The energy intake and expenditure was not different between groups, nor was the energy balance.

Body composition analysis at 10 wk and 20 wk HFD, showed a 30–50% increase in overall adiposity in all groups with the increased time on diet. (Fig. 2b) Similarly, when normalized to body weight, the weight of most white adipose depots, (Fig. 2f–i) increased proportionally over this period, but did not differ between groups. However, the epididymal adipose tissue depot did not increase as expected and there was a significant difference in epididymal adipose weight for 10 week males between the LN:LN and LN:OR groups (*p* = 0.02; Suppl. Table 7), and a significant difference in epididymal adipose weight for 20 week males between the LN:OP and LN:OR groups (*p* < 0.001; Fig. 2d, Suppl. Table 8). In addition, the liver and subscapular brown adipose (Fig. 2c, e) of the LN:OR grew more over the 10–20 week period than the LN:OP group, suggesting the properties of these tissues are altered.

**Fig. 3:**
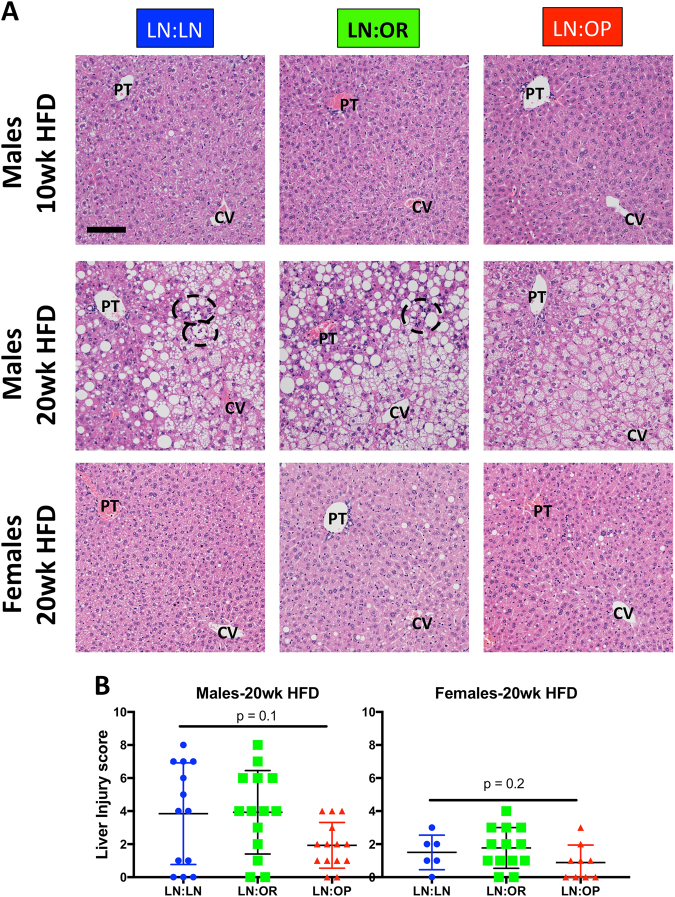
Liver histology/pathology of cross-fostered weanlings after 10 weeks (males only) or 20 weeks (males and females) of HFD. **a** Shown are representative H&E stained sections of LN:LN, LN:OR, or LN:OP mice. The portal triad (PT) and central vein (CV) are indicated. Foci of inflammation are circled. Bar is 100 microns. **b** Liver Injury Scoring of 20 wk HFD for male and female offspring. Scores were generated for individual animals. The mean and standard error of the mean are given, with significance indicated by asterisks. Inflammation included: lobular inflammation, foci of inflammatory cells, lipogranulomas, portal inflammation, Ceroid laden Kupffer cells, Langhans giant cells, foamy macrophages. Steatosis included: macrovesicular steatosis (CLD larger than nuclei), microvesicular steatosis (filled with CLDs, central nucleus). We looked for but did not find any true cell injury (including ballooned cells, acidophil bodies, necrotic cells, hepatocyte megamitochondria, sinusoidal congestion, or abscesses). We also looked for hepatocyte reactive changes indicating a reaction of the liver to the stresses present (we looked for Mallory’s hyaline, glycogenated nuclei, ductal reaction, mitotic figures, hyperplastic nodular regenerative pattern, giant cell formation and large increases in polyploidization) but only saw a very mild ductular reaction in a few of the livers

In order to identify HFD-induced pathology, harvested tissue sections were stained with hematoxylin and eosin. The livers were scored for altered liver histology and injury using a Brunt liver scoring system^17^, modified for mouse tissue^18^, judging various histologic morphologies for presence and quantity. (Suppl. File S4) Because this scoring system is not continuous, we performed a rank-based, nonparametric, Kruskall-Wallis test to see if the overall liver injury score was different. (Males: chi-squared 4.45, df = 2, *p* = 0.108, Females: chi-squared 3.11, df = 2, *p* = 0.211) After 20 weeks of HFD, males had accumulated lipid within the hepatocytes. LN:LN and LN:OR showed both macrosteatosis and microsteatosis, however, LN:OP showed predominantly microsteatosis. (Fig. 3, Suppl. File S4) We also observed a difference in the extent of inflammation. LN:OP males exhibited less inflammation in their livers than LN:LN or LN:OR males. (Fig. 3, Suppl. File S4) Liver cell injury and reactive changes were not seen in our tissues. None of these liver pathologies were observed in males after 10 weeks HFD, nor in females after 20 weeks HFD (Fig. 3a, b)

.

**Fig. 4:**
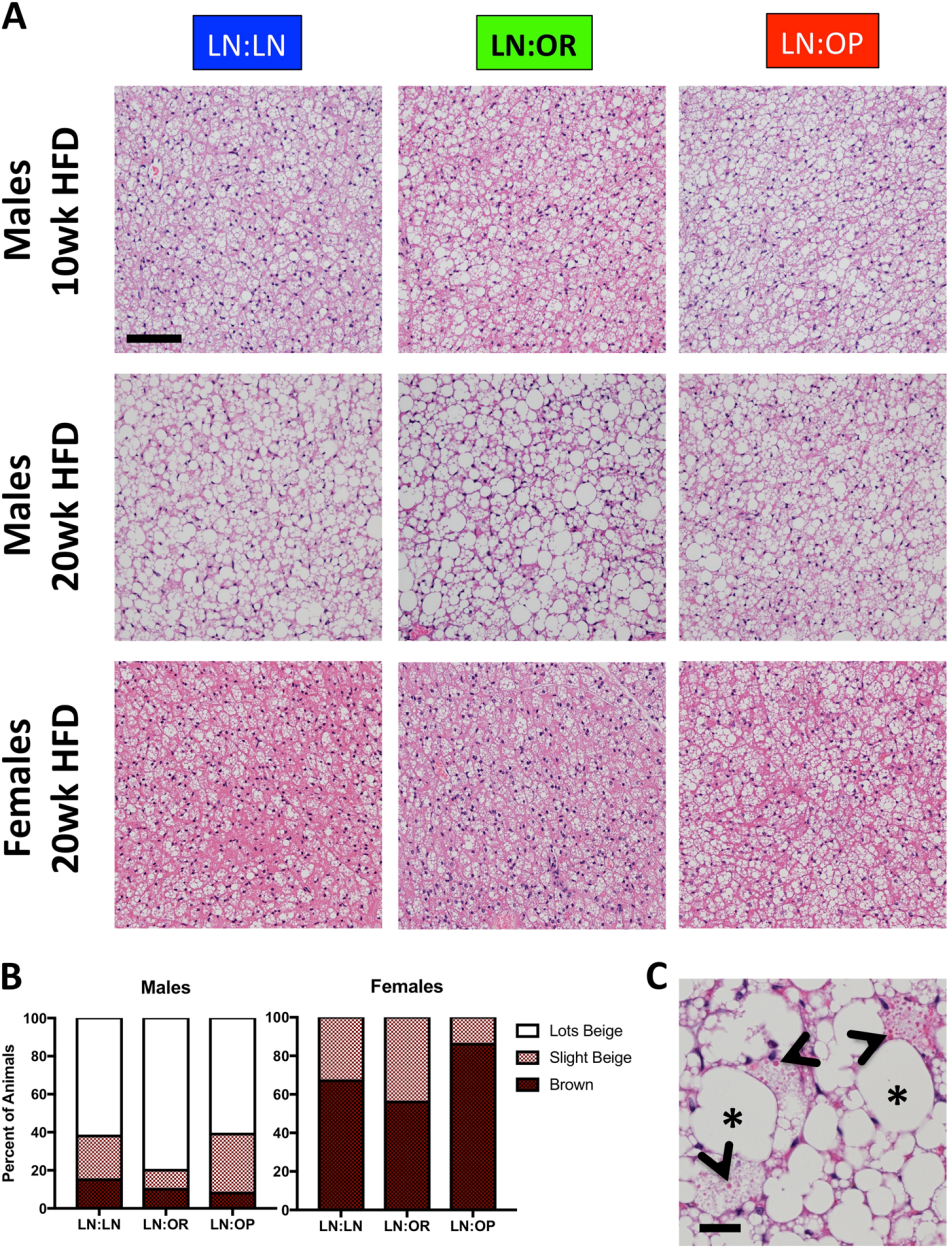
Subscapular BAT histology/pathology of cross-fostered weanlings after 10 weeks (males only) or 20 weeks (males and females) of HFD. **a** Shown are H&E stained sections of LN:LN, LN:OR, or LN:OP mice. Bar is 100 microns. **b** Histological scoring of individual 20 wk HFD male and female mice in each group using the following criteria: Entirely Brown adipose tissue (brown), Some Beige (beige), or Lots of Beige (white). Half of the subscapular BAT was embedded in paraffin. One slice of this tissue depot was surveyed in its entirety to score each animal. Shown are the percent of animals exhibiting above characteristics. **c** Example of megamitochondria in subscapular BAT (arrowheads), as well as large, unilocular lipid droplets (asterisks) indicating significant lipid accumulation or beiging. Bar is 20 um

Since the subscapular BAT responds to metabolic changes, we analyzed the histology of this depot as well. (Fig. 4) Accumulation of excess lipid in the depot, as evidence by lipid droplets several fold larger than the nuclei, was seen in all groups, however, it was most pronounced in the LN:OR group, which had the biggest gain in the weight of this tissue from 10–20 wk HFD. (Fig. 2e) By 20 weeks HFD, most of the male offspring, in all groups, histologically showed loss of true brown adipocytes in this depot. Megamitochondria, an indicator of mitochondrial dysfunction, were present in some of the adipocytes, in most of the animals in both LN:OR and LN:OP groups (64 and 54% respectively), but only 15% of the LN:LN animals. (Fig. 4c) Histological scoring of true brown vs. beige adipose in this depot revealed that females do not accumulate excess lipid in their subscapular BAT (Fig. 4b)

.

**Fig. 5:**
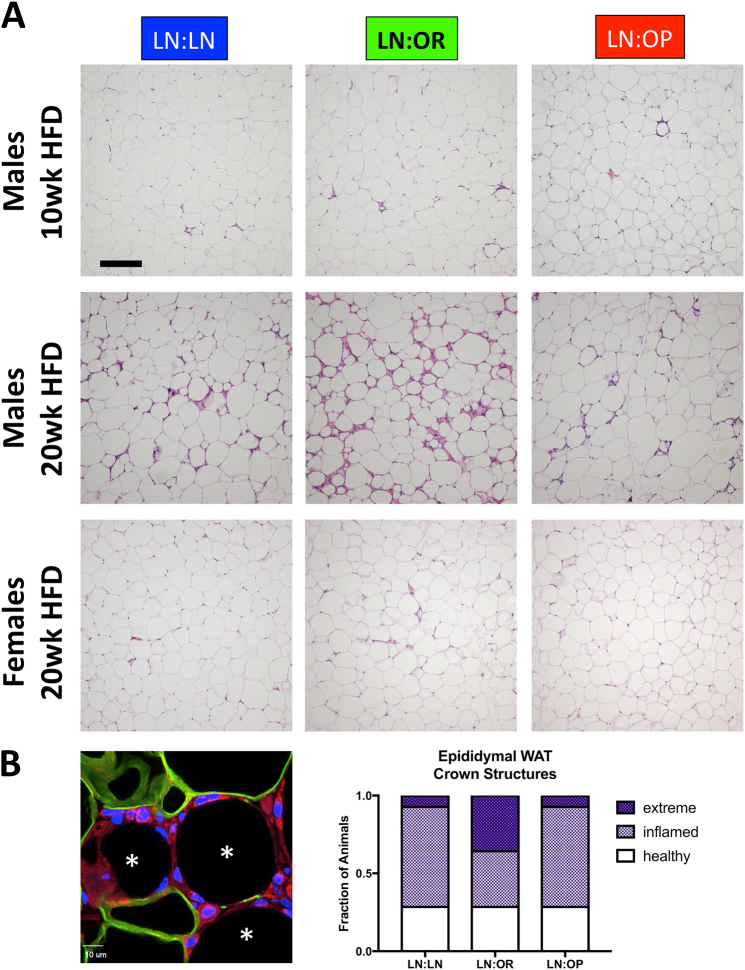
Visceral WAT histology/pathology of cross-fostered weanlings after 10 weeks (Epididymal, males only) or 20 weeks (Epididymal vs. Uterine, males and females) of HFD. **a** Shown are H&E stained sections of LN:LN, LN:OR, or LN:OP mice. Bar is 200 microns. **b** Epididymal WAT sections from 20 week HFD animals were stained with antibody to perilipin 1 (Plin1, green), wheat germ agglutinin (WGA, red), and 4′,6-diamidine-2′-phenylindole dihydrochloride (DAPI, blue). Adipocytes surrounded by WGA-stained inflammatory cells and lacking perilipin-1 surrounding the lipid droplet were counted as dead adipocyte “crown structures”. (asterisks) Shown are crown structure scoring for the visceral WAT of individual offspring males after 20 weeks of HFD, with "extreme" indicating >8 crown structures/field, "inflamed" being 4-8 crown structures/field, and "healthy" WAT showing <4 dead adipocytes/field

We also examined the histology of the epididymal WAT (uterine WAT in females) and noted an increase in crown structures and inflammatory cells in the LN:OR males (Fig. 5). To count these structures, we stained paraffin sections with an antibody to perilipin-1 to score the number of adipocytes with lost integrity per 40 × field. We saw more adipose death in the LN:OR group. One third of the animals in this group had greater than 8 dead adipocytes per field, an extreme level of cell death. Only one animal out of 15 showed this level of inflammation in the LN:LN and LN:OP males. There was, however, large variability, within each group; 1/3 of the animals showed low levels of inflammation, with fewer than 4 dead adipocytes per field. Gene expression analysis in this depot, of a macrophage marker, F4/80, a marker of tissue inflammation (TNF-α), a marker of tissue fibrosis, collagen-1 (Col1a), and tissue inhibitor of metalloproteinase-1 (Timp1) showed an increased (but not statistically significant) expression in the LN:OR group (data not shown). Altogether, these data suggest increased epididymal adipose inflammation in the LN:OR group. To assess whether other adipose depots showed similar levels of inflammation, we subjected H&E stained sections to histopathological scoring. (Suppl. Fig. S3) By 20 weeks HFD, inflammation was present in all depots, with the highest levels seen in the epididymal, perirenal, and mesenteric WAT depots, possibly indicating an overall inflammatory state in all groups of male offspring.

In contrast, the female offspring seem to be protected from adverse metabolic and inflammatory outcomes. We staged and harvested all female offspring in metestrus to avoid hormone-induced differences in metabolic and inflammatory state^27–29^. There was no difference after 20 weeks in body weight or body fat percentage between female offspring nursed on LFD-LN, HFD-OR, or HFD-OP dams, and normalized tissue weights were also not different. Despite food consumption not being different from males, the rate of post-pubertal weight gain and adiposity after 20 weeks HFD was significantly lower, and adipose depot weights differed (Suppl. Fig. S2). Histological analysis of livers from female offspring showed no indication of lipid accumulation or inflammation and less liver injury (Fig. 3b). Subscapular BAT in the females showed minimal beiging (Fig. 4). The uterine WAT depot (compared to the epididymal WAT in the males) showed minimal crown structures, indicating no inflammation (Fig. 5). Histopathological scoring of inflammation was lower in the females for all depots studied (Suppl. Fig. S3).

## Discussion

Other studies of offspring have demonstrated adverse outcomes in those animals born to and nursed on obese dams on a high fat diet. (reviewed in ^30–36^) These previous studies could not differentiate the effects of the diet from the effects of the mother’s adiposity, nor could they isolate the insult to the postnatal period. In order to study whether the mother’s metabolism programs long-term outcomes in the offspring, specifically by transfer through the milk, we studied only pups with no prenatal insult, born to LFD-LN dams. We cross fostered these pups onto DIO dams with variable weight gain so that we compared weight-matched dams on different diets (LFD-LN vs. HFD-OR) and diet-matched dams of different weights (HFD-OR vs. HFD-OP).

Surprisingly, we found the worst outcomes in male pups nursed on dams which resisted high fat diet-induced obesity (HFD-OR). These cross-fostered male pups gained significantly more weight on a HFD in the post-pubertal period, and demonstrated increased pathology and inflammation, over their counterparts nursed on obesity-prone dams (HFD-OP). The protected males and all females showed slower overall weight gain, without a measurable difference in food intake, suggesting a correlation between rate of weight gain and development of pathology. Male pups nursed on HFD-OR dams had increased levels of liver injury, including lipid accumulation, inflammatory foci, and hyperplastic bile ducts, a potentially pre-cancerous change^37^. This group had several animals that demonstrated an extremely high number of crown structures in their epididymal adipose tissue. This depot appeared to be severely inflamed and losing lipid stores, leading to ectopic accumulation of lipid in the liver and subscapular brown adipose tissue. The male pups nursed on LFD-LN and HFD-OP dams showed some, but not all, of these changes to their livers and adipose depots, suggesting that development of these pathologies is occurring at a slower rate.

Milk from mid-lactation was collected and analyzed for differences that could explain the differences in outcome we observed. We found a decrease in the total lipid content in the milk of obesity-prone dams, and a change in the fatty acid composition of this lipid in dams on a high fat diet. Combined, these changes in milk composition result in the delivery of more bioactive fatty acids, which are precursors of inflammatory lipids, to the offspring of obesity-resistant dams on a high fat diet (HFD-OR), during the immediate postnatal period of development. In human infants, perinatal exposures to a high AA/EPA + DHA ratio during the first 4 months of life correlates with infant adiposity^16^.

All mice were weaned onto a 60% fat diet composed of 10:1 lard:soybean oil. Our composition analysis of this diet showed that it was roughly 25% linoleic acid (18:2 n-6), a PUFA, and precursor of arachidonic acid (AA, 20:4 n-6) and other inflammatory lipids thought to promote/prolong inflammation^38^. Indeed, all male offspring developed signs of metabolic dysfunction as a result of consuming this diet. However, the added insult of inflammatory FA in the milk received by the LN:OR group, perhaps programmed more rapid development of metabolic dysfunction. More rapid weight gain in this group, perhaps led to an “unhealthy” expansion of adipose tissue, initiating a system-wide inflammatory state^39^.

The reason for more rapid weight gain in the LN:OR group is unclear. Kojima et al^4^. showed that pups nursing on dams eating HFD seek solid food earlier, which may be mirrored in our pups since LN:LN males were smaller at weaning. Liang et al.^4^ showed that BAT function is impaired in male weanlings nursed on dams on a HFD. These studies would need to be repeated in our model to discern how/if an obesity-prone dam protects her offspring from these outcomes. Buonfiglio et al.^39^ showed that HFD-induced obesity induces a state of prolactin resistance in the dam with direct effects of leptin on the hypothalamus and mammary gland, likely influencing downstream milk production. Buonfiglio’s study did not discern between HFD-fed dams with different levels of obesity. In our study, litters nursed on obese dams on a HFD (HFD-OP) grew 0.3 g/day more slowly than those nursed on lean dams on a HFD (HFD-OR), suggesting that prolactin resistance might be dependent upon or otherwise related to mother’s adiposity. Decreased milk production, leading to decreased neonatal growth, might protect against HFD-induced pathology of nursed offspring later in life.

Although differences in the milk provided to male and female offspring have been documented in humans, non-human primates, cows, deer, voles, and marsupials, these differences seem to be programmed by the fetus in utero, by influencing the development of the mammary gland^43^. Presumably these effects would be muted in the rodent, which carries litters of evenly distributed sexes. Thus, we have no indication that male and female pups received different milk from the same dam. The sex differences seen in offspring outcome is likely an inherent difference in metabolism due to the effects of sex hormones on muscle and adipose fuel utilization, rather than differences in milk consumed^44–47,28,48,49^.

In a study of milk cortisol, Grey et al. conclude, “Mothers have the ability to shape offspring phenotype through the transmission of biologically active components in milk.”^50^ Bioactive substances in milk that affect the development of the neonate have been termed lactocrine factors, or “lactogens”. Included in this list are adipokines, cytokines, orexigenic and anorexigenic stimuli, precursors of inflammatory lipid species, etc. Although milk levels of these factors are rarely measured in mouse models due to small samples sizes, adipokine levels in the mother have been shown to change long-term disease risk in the offspring^51,52^. However, a meta-analysis of disease risk transfer from mother to offspring in rodents, demonstrated that adverse dietary conditions which are present in both pregnancy and lactation carry the worst outcome in offspring^53^, however the mechanisms of this developmental programming are yet unknown. Our work shows that uncharacterized lactogens are present in milk that influence developing metabolic systems, that the mother’s metabolism as well as her diet influence these components of her milk, and that lactation might be a window of susceptibility where intervention is possible.

## Electronic supplementary material

Suppl. Tables 1, 2, 3, 4(JPG 1008 kb)

Figure S1(JPG 795 kb)

Suppl. Tables 5, 6, 7, 8(JPG 1299 kb)

Table S1(JPG 1002 kb)

Figure S4(PDF 91 kb)

Figure S2(JPG 1009 kb)

Suppl. Tables 12, 13, 14(JPG 1278 kb)

Figure S3(JPG 660 kb)

Suppl. Tables 15, 16,17(JPG 1035 kb)
