# Supplementary figures and images for: Maternal obesity during lactation may protect offspring from high fat diet-induced metabolic dysfunction
Source: Nutr Diabetes. 2018 Apr 25;8:18. doi: 10.1038/s41387-018-0027-z (PMC5916951; doi:10.1038/s41387-018-0027-z)

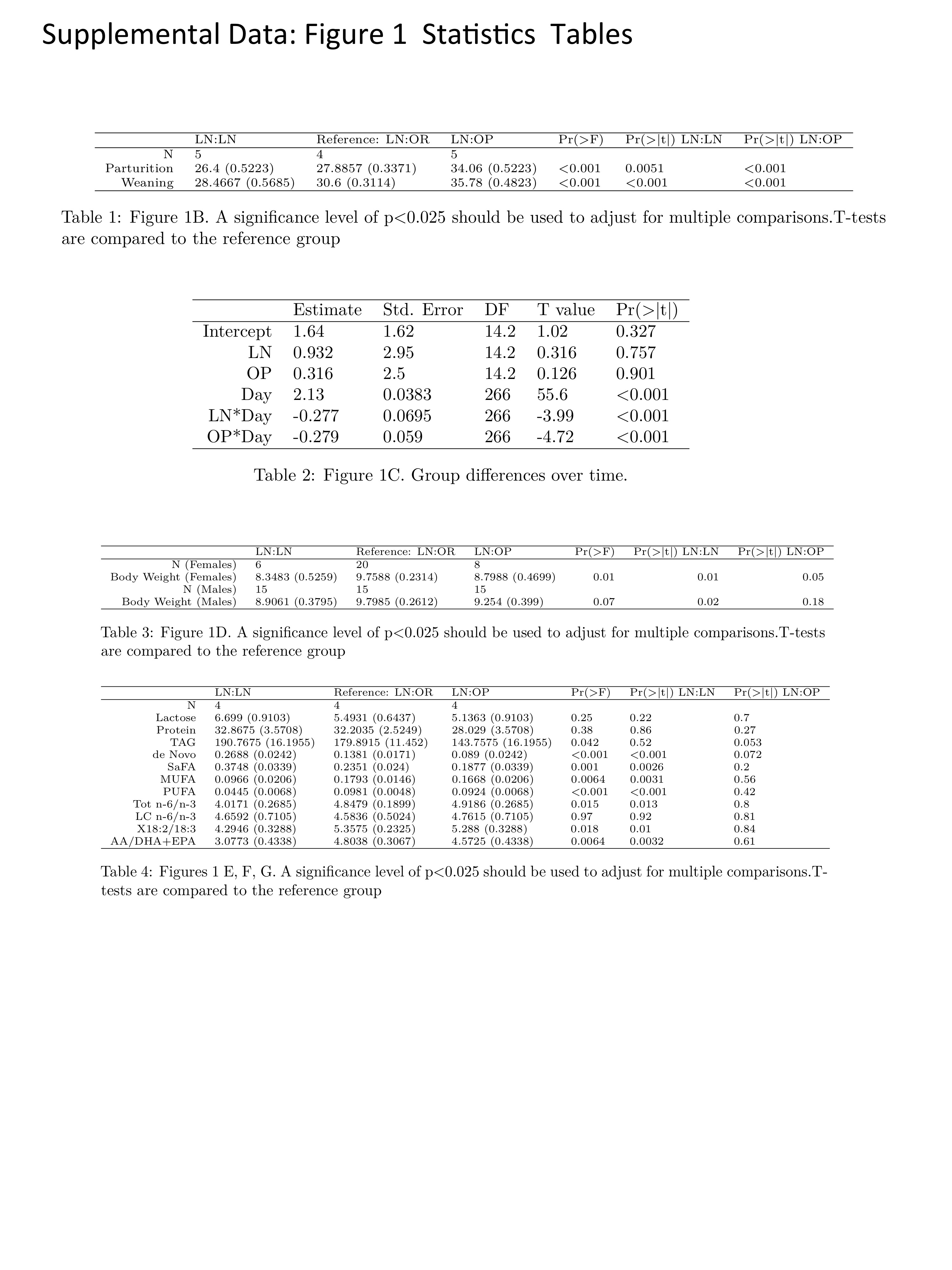

Supplement: Supplementary file 1 — Suppl. Tables 1, 2, 3, 4(JPG 1008 kb) [file 41387_2018_27_MOESM1_ESM.jpg]

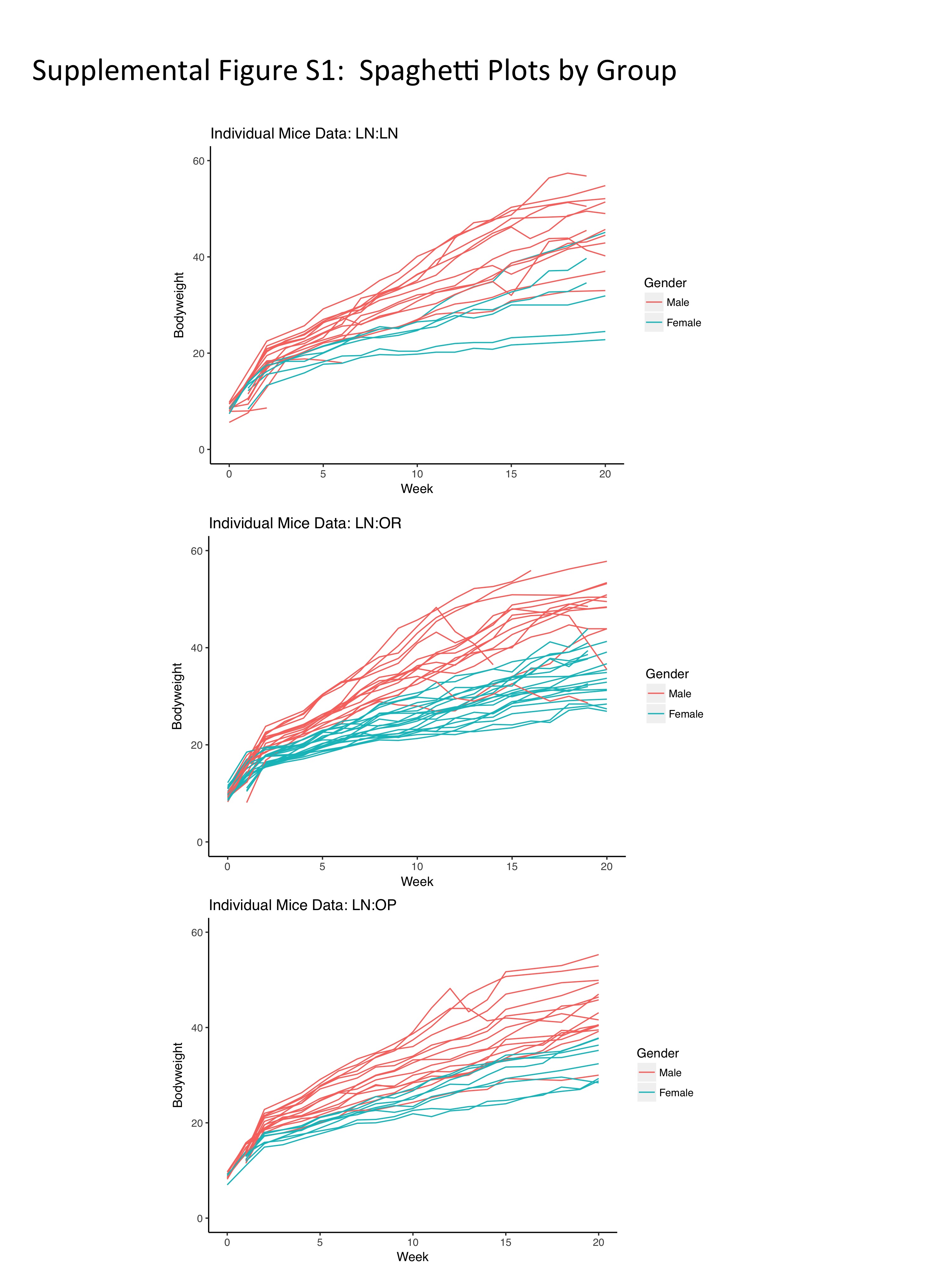

Supplement: Supplementary file 2 — Figure S1(JPG 795 kb) [file 41387_2018_27_MOESM2_ESM.jpg]

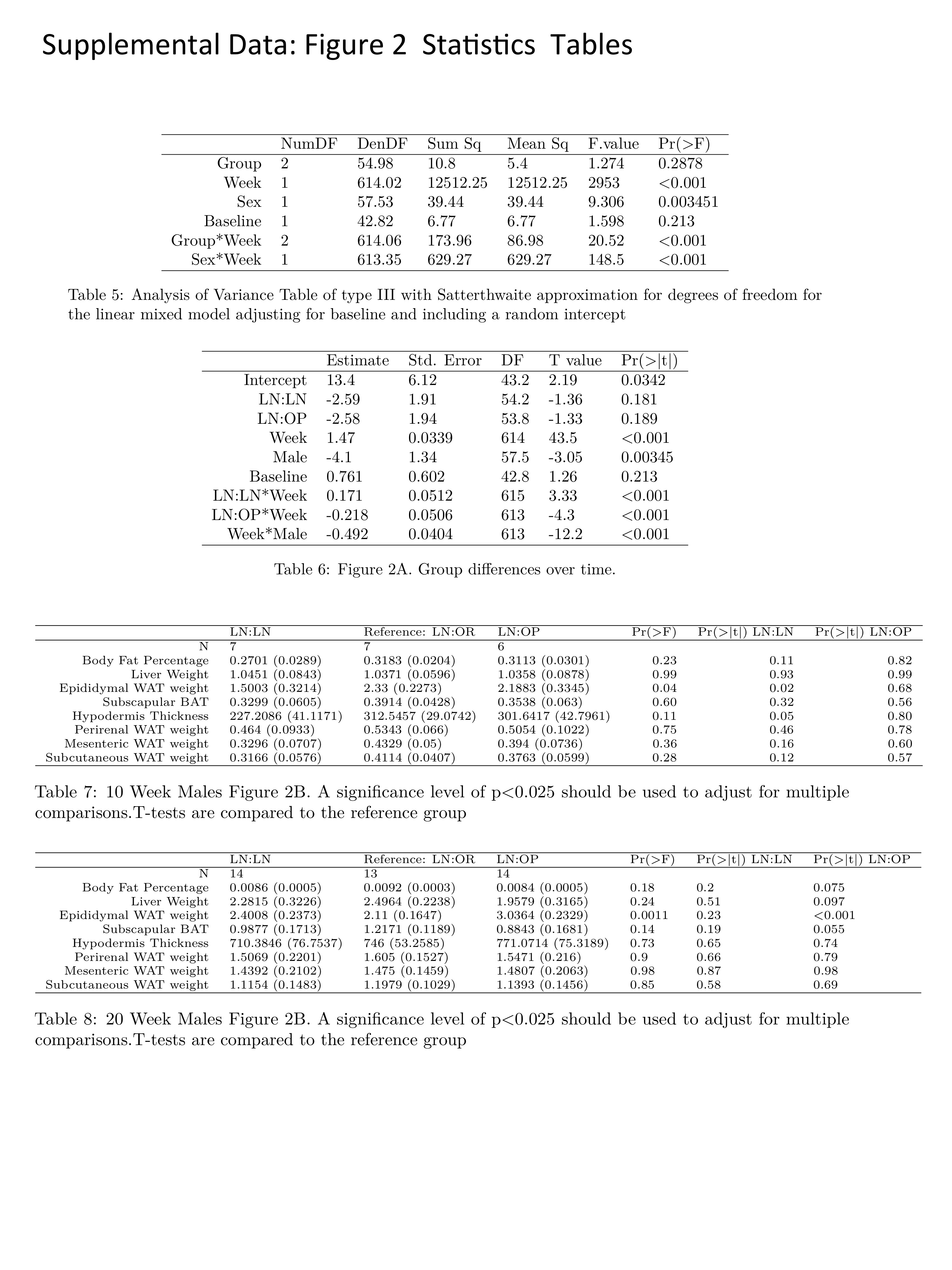

Supplement: Supplementary file 3 — Suppl. Tables 5, 6, 7, 8(JPG 1299 kb) [file 41387_2018_27_MOESM3_ESM.jpg]

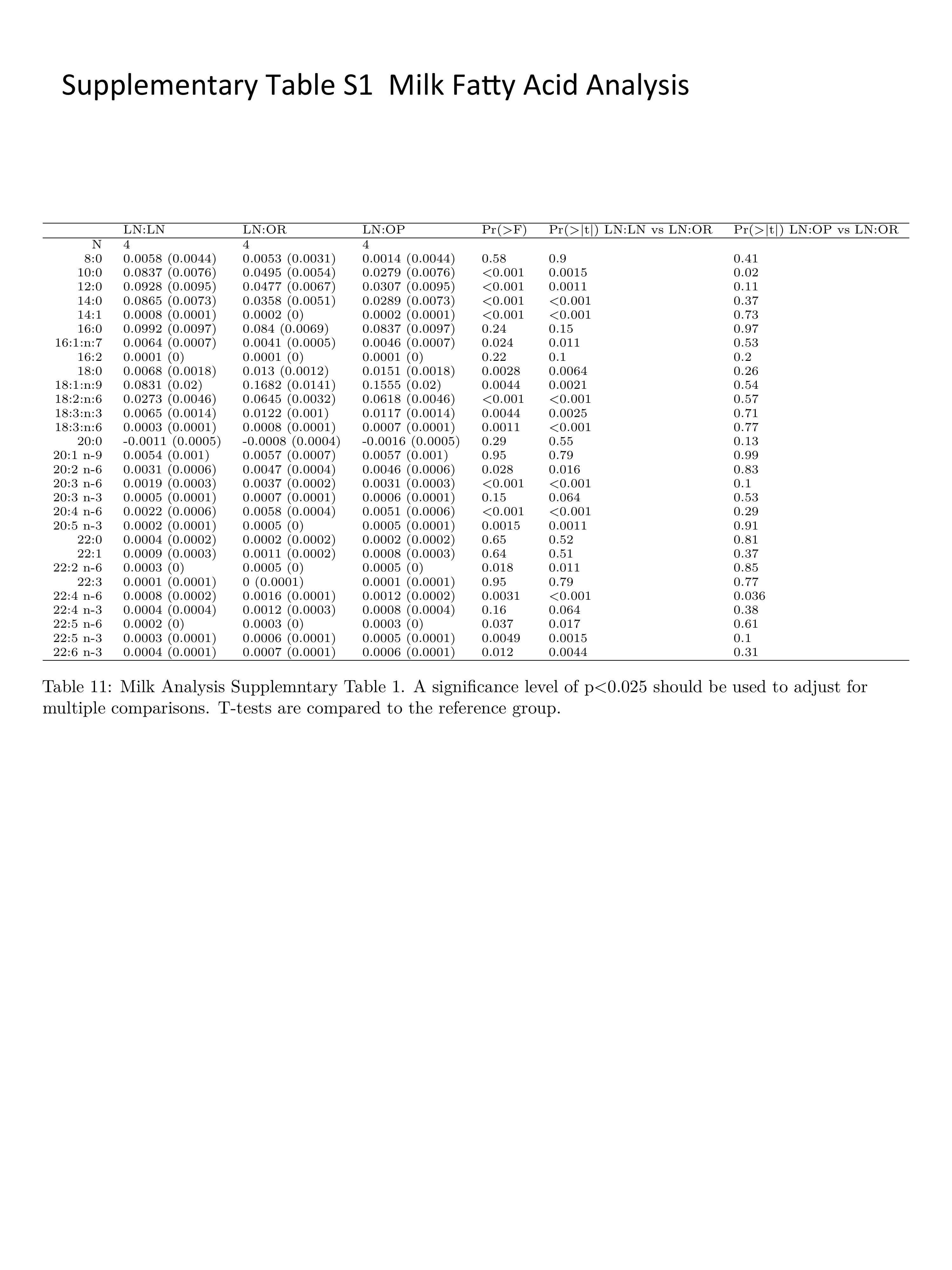

Supplement: Supplementary file 4 — Table S1(JPG 1002 kb) [file 41387_2018_27_MOESM4_ESM.jpg]

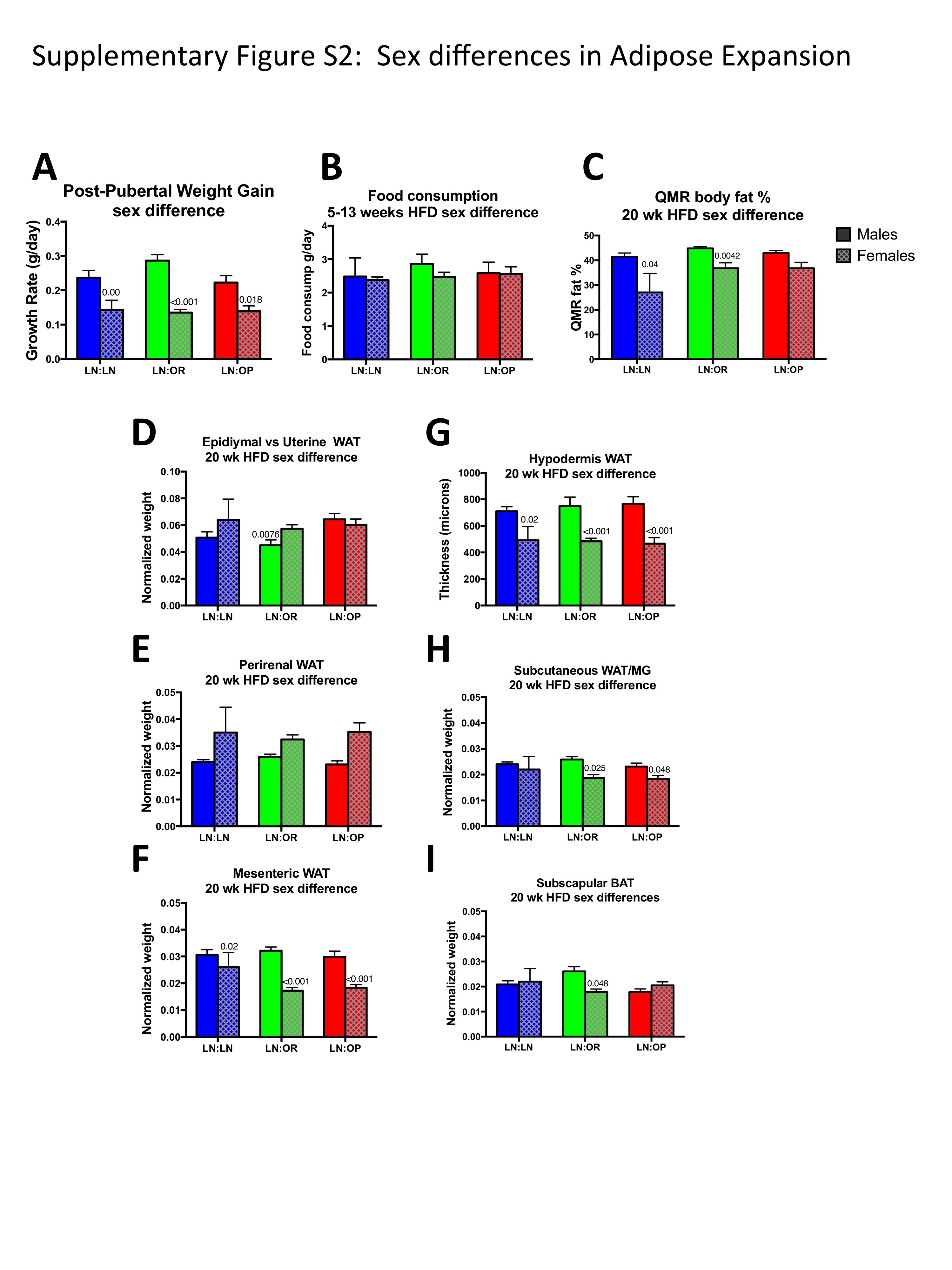

Supplement: Supplementary file 6 — Figure S2(JPG 1009 kb) [file 41387_2018_27_MOESM6_ESM.jpg]

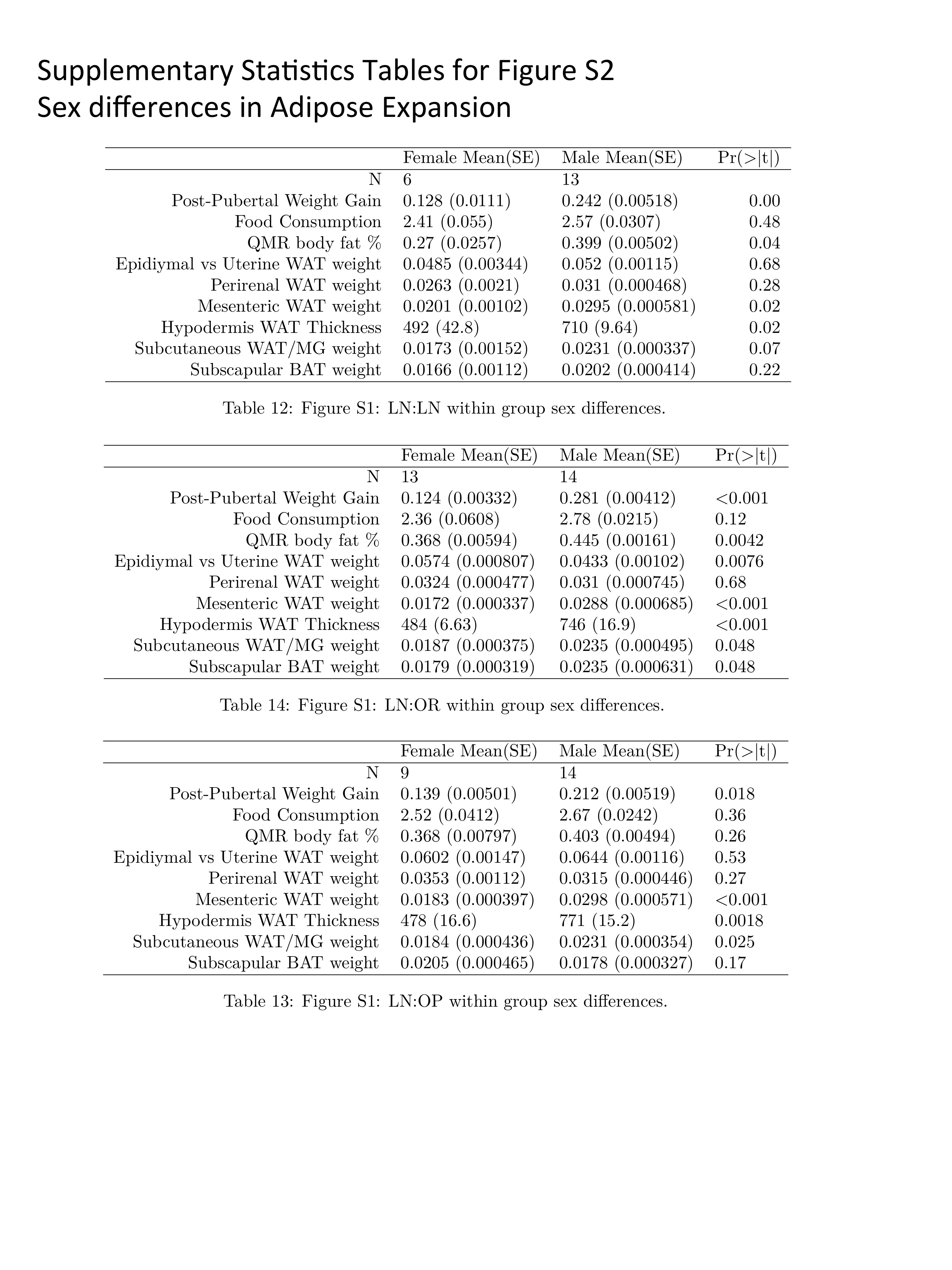

Supplement: Supplementary file 7 — Suppl. Tables 12, 13, 14(JPG 1278 kb) [file 41387_2018_27_MOESM7_ESM.jpg]

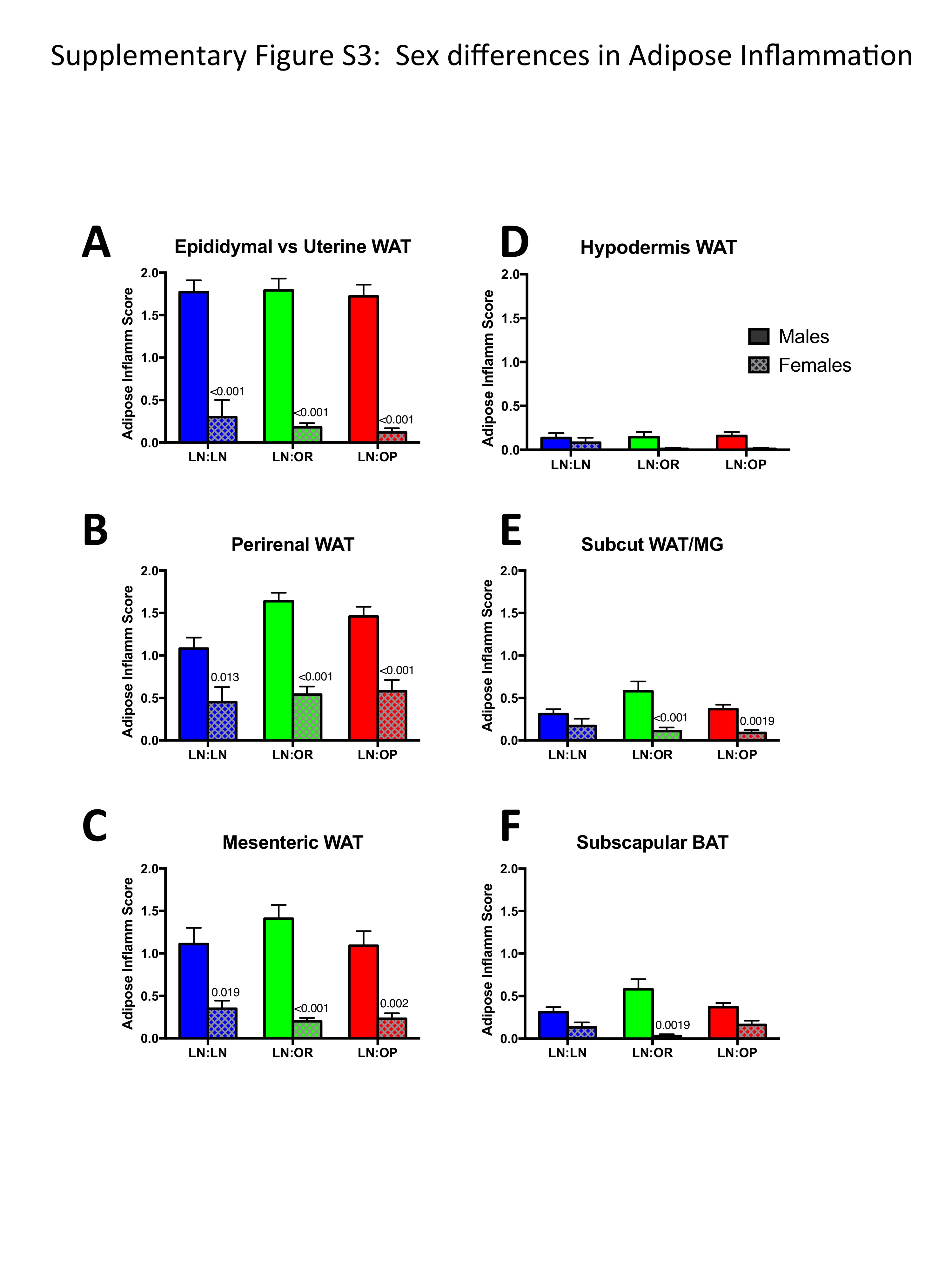

Supplement: Supplementary file 8 — Figure S3(JPG 660 kb) [file 41387_2018_27_MOESM8_ESM.jpg]

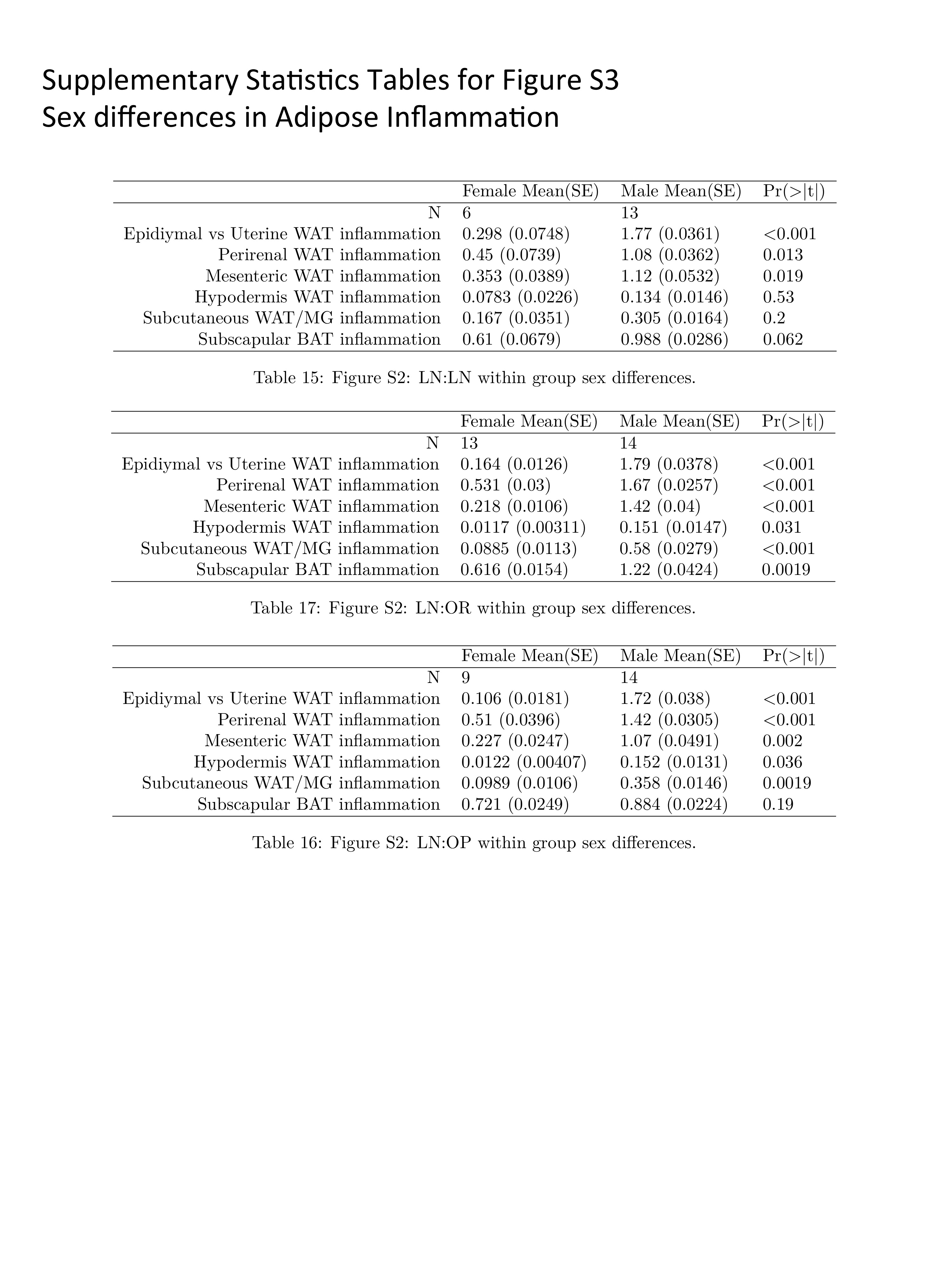

Supplement: Supplementary file 9 — Suppl. Tables 15, 16,17(JPG 1035 kb) [file 41387_2018_27_MOESM9_ESM.jpg]
