# Supplementary material for: Maternal obesity during lactation may protect offspring from high fat diet-induced metabolic dysfunction
Source: Nutr Diabetes. 2018 Apr 25;8:18. doi: 10.1038/s41387-018-0027-z (PMC5916951; doi:10.1038/s41387-018-0027-z)
Supplement: Supplementary file 5 — Figure S4(PDF 91 kb) [file 41387_2018_27_MOESM5_ESM.pdf]

**01/31/2017 Re-Scoring of: 04/05/2015 Brunt Scoring of Monks 20 wk LN OP OR foster pup expt**

H&E for most criteria
PSR for Fibrosis
PAS for foamy macs

| | | | | | | | | | | | | | | | | | | | | | | | | |
|---|---|---|---|---|---|---|---|---|---|---|---|---|---|---|---|---|---|---|---|---|---|---|---|---|
| | Group ---> | | LN:LN | | | | | | | | | | | | | LN:LN | | | | LN:OP | | | | |
| | animal# ---> | | 367 | 367 | 367 | 367 | 367 | 367 | 367 | 367 | 368 | 368 | 368 | 368 | 368 | | | | | 360 | 360 | 360 | 360 | 361 |
| | animal sub # ---> | | LA | LB | LC | LF | LG | LH | RLA | RLB | A | B | C | RA | RC | | | | | LA | LB | LC | LD | RA |
| Feature | | Points | | | | | | | | | | | | | | ave | std dev | N | sem | | | | | |
| **Liver cell injury** | | | | | | | | | | | | | | | | | | | | | | | | |
| ballooning | none | 0 | 0 | 0 | 0 | 0 | 0 | 0 | 0 | 0 | 0 | 0 | 0 | 0 | 0 | | | | | 0 | 0 | 0 | 0 | 0 |
| | few | 2 | | | | | | | | | | | | | | | | | | | | | | |
| | many | 4 | | | | | | | | | | | | | | | | | | | | | | |
| acidophil bodies | none | 0 | 0 | 0 | 0 | 0 | 0 | 0 | 0 | 0 | 0 | 0 | 0 | 0 | 0 | | | | | 0 | 0 | 0 | 0 | 0 |
| | few | 2 | | | | | | | | | | | | | | | | | | | | | | |
| | many (≥1 per 200x field) | 4 | | | | | | | | | | | | | | | | | | | | | | |
| Necrotic cells | none | 0 | 0 | 0 | 0 | 0 | 0 | 0 | 0 | 0 | 0 | 0 | 0 | 0 | 0 | | | | | 0 | 0 | 0 | 0 | 0 |
| | present (≥2/200x field) | 2 | | | | | | | | | | | | | | | | | | | | | | |
| pigmented macrophages | none | 0 | 0 | 0 | 0 | 0 | 0 | 0 | 0 | 0 | 0 | 0 | 0 | 0 | 0 | | | | | 0 | 0 | 0 | 0 | 0 |
| | many | 1 | | | | | | | | | | | | | | | | | | | | | | |
| megamitochondria | none | 0 | 0 | 0 | 0 | 0 | 0 | 0 | 0 | 0 | 0 | 0 | 0 | 0 | 0 | | | | | 0 | 0 | 0 | 0 | 0 |
| | present | 1 | | | | | | | | | | | | | | | | | | | | | | |
| sinusoidal dilatation & congest | none | 0 | 0 | 0 | 0 | 0 | 0 | 0 | 0 | 0 | 0 | 0 | 0 | 0 | 0 | | | | | 0 | 0 | 0 | 0 | 0 |
| | Yes (≥3 RBC diam) | 1 | | | | | | | | | | | | | | | | | | | | | | |
| True abscesses (>6 cell diams) | no | 0 | 0 | 0 | 0 | 0 | 0 | 0 | 0 | 0 | 0 | 0 | 0 | 0 | 0 | | | | | 0 | 0 | 0 | 0 | 0 |
| | Yes (>1 / 40x field) | 2 | | | | | | | | | | | | | | | | | | | | | | |
| **Injury Subtotal** | | | **0** | **0** | **0** | **0** | **0** | **0** | **0** | **0** | **0** | **0** | **0** | **0** | **0** | **0** | **0** | **13** | **0** | **0** | **0** | **0** | **0** | **0** |
| **Inflammation** | | | | | | | | | | | | | | | | | | | | | | | | |
| lobular inflammation | <2 per 200x field | 0 | 0 | 0 | 0 | | 0 | | | | 0 | | 0 | | 0 | | | | | 0 | 0 | 0 | 0 | 0 |
| | 2 per 200x field | 1 | | | | | | | | 1 | | 1 | | 1 | | | | | | | | | | |
| | 2-4 per 200x field | 2 | | | | 2 | | 2 | 2 | | | | | | | | | | | | | | | |
| | >4 per 200x field | 3 | | | | | | | | | | | | | | | | | | | | | | |
| foci of inflammatory cells | absent ( < 1 / 200x field) | 0 | 0 | | 0 | | 0 | | | | 0 | | 0 | | | | | | | 0 | 0 | | 0 | |
| | present (1 or more / 200x field) | 1 | | 1 | | 1 | | 1 | 1 | 1 | | 1 | | 1 | 1 | | | | | | | 1 | | 1 |
| lipogranulomas | absent | 0 | 0 | | 0 | | 0 | | | | 0 | 0 | 0 | | | | | | | 0 | 0 | 0 | 0 | |
| | present | 2 | | 1 | | 1 | | 1 | 1 | 1 | | | | 1 | 1 | | | | | | | | | 1 |
| portal inflammation | none to minimal | 0 | 0 | 0 | 0 | 0 | 0 | 0 | 0 | 0 | 0 | 0 | 0 | 0 | 0 | | | | | 0 | 0 | 0 | 0 | 0 |
| | more than minimal | 1 | | | | | | | | | | | | | | | | | | | | | | |
| Ceroid laden Kupffer c | No | 0 | 0 | | 0 | | 0 | | | | 0 | | 0 | | 0 | | | | | | 0 | | 0 | 0 |
| | Yes (≥3 / 200x field) | 1 | | 1 | | 1 | | 1 | 1 | 1 | | 1 | | 1 | | | | | | 1 | | 1 | | |
| Langhans giant cells | No | 0 | 0 | 0 | 0 | 0 | 0 | 0 | 0 | 0 | 0 | 0 | 0 | 0 | 0 | | | | | 0 | 0 | 0 | 0 | 0 |
| | Yes (≥1 / 200x field) | 1 | | | | | | | | | | | | | | | | | | | | | | |
| Foamy macrophages | No | 0 | 0 | 0 | 0 | 0 | 0 | 0 | 0 | 0 | 0 | 0 | 0 | 0 | 0 | | | | | 0 | 0 | 0 | 0 | 0 |
| | Yes | 1 | | | | | | | | | | | | | | | | | | | | | | |
| **Inflam Subtotal** | | | **0** | **3** | **0** | **5** | **0** | **5** | **5** | **4** | **0** | **3** | **0** | **4** | **2** | **2** | **2** | **13** | **1** | **1** | **0** | **2** | **0** | **2** |
| **Reactive Chnages** | | | | | | | | | | | | | | | | | | | | | | | | |
| Mallory's hyaline | none | 0 | 0 | 0 | 0 | 0 | 0 | 0 | 0 | 0 | 0 | 0 | 0 | 0 | 0 | | | | | 0 | 0 | 0 | 0 | 0 |
| | many | 1 | | | | | | | | | | | | | | | | | | | | | | |
| Glycogenated nuclei | none ( < 1 / 200x field) | 0 | 0 | 0 | 0 | 0 | 0 | 0 | 0 | 0 | 0 | 0 | 0 | 0 | 0 | | | | | 0 | 0 | 0 | 0 | 0 |
| | few (1-3 per 200x field) | 1 | | | | | | | | | | | | | | | | | | | | | | |
| | many ( > 3 /200x field) | 2 | | | | | | | | | | | | | | | | | | | | | | |
| ductal reaction | none | 0 | 0 | 0 | 0 | | 0 | 0 | 0 | 0 | 0 | 0 | 0 | | 0 | | | | | 0 | 0 | 0 | 0 | 0 |
| | minor | 1 | | | | 1 | | | | | | | | 1 | | | | | | | | | | |
| | robust | 2 | | | | | | | | | | | | | | | | | | | | | | |
| mitotic figures | <2 per 200x field | 0 | 0 | 0 | 0 | 0 | 0 | 0 | 0 | 0 | 0 | 0 | 0 | 0 | 0 | | | | | 0 | 0 | 0 | 0 | 0 |
| | >2 per 200x field | 0 | | | | | | | | | | | | | | | | | | | | | | |
| hyperpl nod regen morph | no | 0 | 0 | 0 | 0 | 0 | 0 | 0 | 0 | 0 | 0 | 0 | 0 | 0 | 0 | | | | | 0 | 0 | 0 | 0 | 0 |
| | yes | 1 | | | | | | | | | | | | | | | | | | | | | | |
| hyalinized & thickened PV&HA | none | 0 | 0 | 0 | 0 | 0 | 0 | 0 | 0 | 0 | 0 | 0 | 0 | 0 | 0 | | | | | 0 | 0 | 0 | 0 | 0 |
| | present | 1 | | | | | | | | | | | | | | | | | | | | | | |
| Giant Cell Transformation | No | 0 | 0 | 0 | 0 | 0 | 0 | 0 | 0 | 0 | 0 | 0 | 0 | 0 | 0 | | | | | 0 | 0 | 0 | 0 | 0 |
| | Yes | 1 | | | | | | | | | | | | | | | | | | | | | | |
| **Reactive Subtotal** | | | **0** | **0** | **0** | **1** | **0** | **0** | **0** | **0** | **0** | **0** | **0** | **1** | **0** | **0** | **0** | **13** | **0** | **0** | **0** | **0** | **0** | **0** |
| **Steatosis** | | | | | | | | | | | | | | | | | | | | | | | | |
| Macrovesicular steatosis | <10% | 0 | 0 | | 0 | | 0 | | | | 0 | 0 | 0 | | | | | | | 0 | 0 | 0 | | |
| (CLD larger then nuclei) | 10-33% | 1 | | 1 | | 1 | | 1 | 1 | 1 | | | | 1 | 1 | | | | | | | | 1 | 1 |
| | 33-66% | 2 | | | | | | | | | | | | | | | | | | | | | | |
| | >66% | 3 | | | | | | | | | | | | | | | | | | | | | | |
| Microvesicular steatosis | not present | 0 | | | | | 0 | | | | 0 | | 0 | | | | | | | | | | | |
| (filled with CLDs, central nuc | present (>5% of hepatos) | 1 | 1 | 1 | 1 | 1 | | 1 | 1 | 1 | | 1 | | 1 | 1 | | | | | 1 | 1 | 1 | 1 | 1 |
| **Steatosis Subtotal** | | | **1** | **2** | **1** | **2** | **0** | **2** | **2** | **2** | **0** | **1** | **0** | **2** | **2** | **1** | **1** | **13** | **0** | **1** | **1** | **1** | **2** | **2** |
| **Fibrosis** | | | | | | | | | | | | | | | | | | | | | | | | |
| **now assessed by quantitative PSR stn** | | | **now assessed by quantitative PSR stn** | | | | | | | | | | | | | | | | | **now assessed by quantitative PSR** | | | | |
| (see separate graphed result) | | | | | | | | | | | | | | | | | | | | | | | | |

**Summary**

| | | | | | | | | | | | | | | | | | | | | | |
|---|---|---|---|---|---|---|---|---|---|---|---|---|---|---|---|---|---|---|---|---|---|
| | LN:LN | | | | | | | | | | | | | LN:LN | | | | LN:OP | | | | |
| | 367 | 367 | 367 | 367 | 367 | 367 | 367 | 367 | 368 | 368 | 368 | 368 | 368 | | | | | 360 | 360 | 360 | 360 | 361 |
| **Scoring by Category** | LA | LB | LC | LF | LG | LH | RLA | RLB | A | B | C | RA | RC | | | | | LA | LB | LC | LD | RA |
| **Cell Injury** | **0** | **0** | **0** | **0** | **0** | **0** | **0** | **0** | **0** | **0** | **0** | **0** | **0** | **0.00** | **0.00** | **13.00** | **0.00** | **0** | **0** | **0** | **0** | **0** |
| **Inflammation** | **0** | **3** | **0** | **5** | **0** | **5** | **5** | **4** | **0** | **3** | **0** | **4** | **2** | **2.38** | **2.14** | **13.00** | **0.62** | **1** | **0** | **2** | **0** | **2** |
| **Reactive Changes** | **0** | **0** | **0** | **1** | **0** | **0** | **0** | **0** | **0** | **0** | **0** | **1** | **0** | **0.15** | **0.38** | **13.00** | **0.11** | **0** | **0** | **0** | **0** | **0** |
| **Steatosis** | **1** | **2** | **1** | **2** | **0** | **2** | **2** | **2** | **0** | **1** | **0** | **2** | **2** | **1.31** | **0.85** | **13.00** | **0.25** | **1** | **1** | **1** | **2** | **2** |
| **Fibrosis** Not Included Here, Quantitated Separately | | | | | | | | | | | | | | | | | | | | | | |
| **Total** | **1** | **5** | **1** | **8** | **0** | **7** | **7** | **6** | **0** | **4** | **0** | **7** | **4** | | | | | **2** | **1** | **3** | **2** | **4** |
| **ave** | | | | | | | | | | | | | | **3.85** | | | | | | | | |
| **std dev** | | | | | | | | | | | | | | **3.08** | | | | | | | | |
| **n** | | | | | | | | | | | | | | **13** | | | | | | | | |
| **sqr rt N-1** | | | | | | | | | | | | | | **3.46** | | | | | | | | |
| **sem** | | | | | | | | | | | | | | **0.89** | | | | | | | | |

| 361 RB | 361 RC | 356 RLA | 356 RLD | 359 RLA | 359 RLB | 360 RLA | 360 RLB | 360 RLC | ave | std dev | N | sem |
|---|---|---|---|---|---|---|---|---|---|---|---|---|
| 0 | 0 | 0 | 0 | 0 | 0 | 0 | 0 | 0 | | | | |
| 0 | 0 | 0 | 0 | 0 | 0 | 0 | 0 | 0 | | | | |
| 0 | 0 | 0 | 0 | 0 | 0 | 0 | 0 | 0 | | | | |
| 0 | 0 | 0 | 0 | 0 | 0 | 0 | 0 | 0 | | | | |
| 0 | 0 | 0 | 0 | 0 | 0 | 0 | 0 | 0 | | | | |
| 0 | 0 | 0 | 0 | 0 | 0 | 0 | 0 | 0 | | | | |
| 0 | 0 | 0 | 0 | 0 | 0 | 0 | 0 | 0 | | | | |
| **0** | **0** | **0** | **0** | **0** | **0** | **0** | **0** | **0** | | | | |
| 0 | 0 | 0 | 0 | 0 | 0 | 0 | 0 | 0 | | | | |
| 0 | | 0 | 0 | 0 | 0 | 0 | 0 | 0 | | | | |
| | 1 | | | | | | | | | | | |
| 0 | | | 0 | 0 | 0 | 0 | 0 | 0 | | | | |
| | 1 | 1 | | | | | | | | | | |
| 0 | 0 | 0 | 0 | 0 | 0 | 0 | 0 | 0 | | | | |
| | 0 | | | 0 | | 0 | 0 | 0 | | | | |
| 1 | | 1 | | | 1 | | | | | | | |
| 0 | 0 | 0 | 0 | 0 | 0 | 0 | 0 | 0 | | | | |
| 0 | 0 | 0 | 0 | 0 | 0 | 0 | 0 | 0 | | | | |
| **1** | **2** | **2** | **0** | **0** | **1** | **0** | **0** | **0** | | | | |
| 0 | 0 | 0 | 0 | 0 | 0 | 0 | 0 | 0 | | | | |
| 0 | 0 | 0 | 0 | 0 | 0 | 0 | 0 | 0 | | | | |
| 0 | 0 | | 0 | 0 | 0 | 0 | 0 | 0 | | | | |
| | | 1 | | | | | | | | | | |
| 0 | 0 | 0 | 0 | 0 | 0 | 0 | 0 | 0 | | | | |
| 0 | 0 | 0 | 0 | 0 | 0 | 0 | 0 | 0 | | | | |
| 0 | 0 | 0 | 0 | 0 | 0 | 0 | 0 | 0 | | | | |
| 0 | 0 | 0 | 0 | 0 | 0 | 0 | 0 | 0 | | | | |
| **0** | **0** | **1** | **0** | **0** | **0** | **0** | **0** | **0** | | | | |
| 0 | | 0 | 0 | 0 | 0 | 0 | 0 | 0 | | | | |
| | 1 | | | | | | | | | | | |
| | | | | 0 | | | | 0 | | | | |
| 1 | 1 | 1 | 1 | | 1 | 1 | 1 | | | | | |
| **1** | **2** | **1** | **1** | **0** | **1** | **1** | **1** | **0** | | | | |

t stn

| LN:OR | | | | | | | | | | | | | | | | | |
|---|---|---|---|---|---|---|---|---|---|---|---|---|---|---|---|---|---|
| 356 A | 356 B | 358 LA | 358 LB | 359 RA | 360 RA | 360 RC | 359 RB | 359 RC | 359 RF | 359 RG | 359 RH | 364 A | 364 B | ave | std dev | N | sem |
| 0 | 0 | 0 | 0 | 0 | 0 | 0 | 0 | 0 | 0 | 0 | 0 | 0 | 0 | | | | |
| 0 | 0 | 0 | 0 | 0 | 0 | 0 | 0 | 0 | 0 | 0 | 0 | 0 | 0 | | | | |
| 0 | 0 | 0 | 0 | 0 | 0 | 0 | 0 | 0 | 0 | 0 | 0 | 0 | 0 | | | | |
| 0 | 0 | 0 | 0 | 0 | 0 | 0 | 0 | 0 | 0 | 0 | 0 | 0 | 0 | | | | |
| 0 | 0 | 0 | 0 | 0 | 0 | 0 | 0 | 0 | 0 | 0 | 0 | 0 | 0 | | | | |
| 0 | 0 | 0 | 0 | 0 | 0 | 0 | 0 | 0 | 0 | 0 | 0 | 0 | 0 | | | | |
| 0 | 0 | 0 | 0 | 0 | 0 | 0 | 0 | 0 | 0 | 0 | 0 | 0 | 0 | | | | |
| **0** | **0** | **0** | **0** | **0** | **0** | **0** | **0** | **0** | **0** | **0** | **0** | **0** | **0** | | | | |
| 0 | 0 | 0 | 0 | | 0 | | 0 | 0 | 0 | 0 | 0 | | 0 | | | | |
| | | | | 1 | | 1 | | | | | | 1 | | | | | |
| 0 | 0 | 0 | 0 | | 0 | | | | | 0 | 0 | | 0 | | | | |
| | | | | 1 | | 1 | 1 | 1 | 1 | | | 1 | | | | | |
| 0 | 0 | 0 | 0 | | 0 | | | | | 0 | 0 | | | | | | |
| | | | | 1 | | 1 | 1 | 1 | 1 | | | 1 | 1 | | | | |
| 0 | 0 | 0 | 0 | 0 | 0 | 0 | 0 | 0 | 0 | 0 | 0 | 0 | 0 | | | | |
| | 0 | 0 | 0 | | | | 0 | | | 0 | | | | | | | |
| 1 | | | | 1 | 1 | 1 | | 1 | 1 | | 1 | 1 | 1 | | | | |
| 0 | 0 | 0 | 0 | 0 | 0 | 0 | 0 | 0 | 0 | 0 | 0 | 0 | 0 | | | | |
| 0 | 0 | 0 | 0 | 0 | 0 | 0 | 0 | 0 | 0 | 0 | 0 | 0 | 0 | | | | |
| **1** | **0** | **0** | **0** | **4** | **1** | **4** | **2** | **3** | **3** | **0** | **1** | **4** | **2** | | | | |
| 0 | 0 | 0 | 0 | 0 | 0 | 0 | 0 | 0 | 0 | 0 | 0 | 0 | 0 | | | | |
| 0 | 0 | 0 | 0 | 0 | 0 | 0 | 0 | 0 | 0 | 0 | 0 | 0 | 0 | | | | |
| | 0 | 0 | 0 | 0 | | | 0 | | | 0 | | | 0 | | | | |
| 1 | | | | | 1 | 1 | | 1 | 1 | | 1 | 1 | | | | | |
| 0 | 0 | 0 | 0 | 0 | 0 | 0 | 0 | 0 | 0 | 0 | 0 | 0 | 0 | | | | |
| 0 | 0 | 0 | 0 | 0 | 0 | 0 | 0 | 0 | 0 | 0 | 0 | 0 | 0 | | | | |
| 0 | 0 | 0 | 0 | 0 | 0 | 0 | 0 | 0 | 0 | 0 | 0 | 0 | 0 | | | | |
| 0 | 0 | 0 | 0 | 0 | 0 | 0 | 0 | 0 | 0 | 0 | 0 | 0 | 0 | | | | |
| **1** | **0** | **0** | **0** | **0** | **1** | **1** | **0** | **1** | **1** | **0** | **1** | **1** | **0** | | | | |
| | 0 | 0 | | | | | | | | 0 | | | 0 | | | | |
| 1 | | | 1 | 1 | 1 | | 1 | 1 | 1 | | 1 | 1 | | | | | |
| | | | | | | 2 | | | | | | | | | | | |
| | | 0 | | | | | | | | 0 | | | | | | | |
| 1 | 1 | | 1 | 1 | 1 | 1 | 1 | 1 | 1 | | 1 | 1 | 1 | | | | |
| **2** | **1** | **0** | **2** | **2** | **2** | **3** | **2** | **2** | **2** | **0** | **2** | **2** | **1** | | | | |

now assessed by quantitative PSR stn

| | | | | | | | | | LN:OP | | | |
|---|---|---|---|---|---|---|---|---|---|---|---|---|
| **361 RB** | **361 RC** | **356 RLA** | **356 RLD** | **359 RLA** | **359 RLB** | **360 RLA** | **360 RLB** | **360 RLC** | | | | |
| **0** | **0** | **0** | **0** | **0** | **0** | **0** | **0** | **0** | **0.00** | **0.00** | **14.00** | **0.00** |
| **1** | **2** | **2** | **0** | **0** | **1** | **0** | **0** | **0** | **0.79** | **0.89** | **14.00** | **0.25** |
| **0** | **0** | **1** | **0** | **0** | **0** | **0** | **0** | **0** | **0.07** | **0.27** | **14.00** | **0.07** |
| **1** | **2** | **1** | **1** | **0** | **1** | **1** | **1** | **0** | **1.07** | **0.62** | **14.00** | **0.17** |
| **2** | **4** | **4** | **1** | **0** | **2** | **1** | **1** | **0** | | | | |
| | | | | | | | | | **1.93** | | | |
| | | | | | | | | | **1.38** | | | |
| | | | | | | | | | **14** | | | |
| | | | | | | | | | **3.61** | | | |
| | | | | | | | | | **0.38** | | | |

| **LN:OR** | | | | | | | | | | | | | | **LN:OR** | | | |
|---|---|---|---|---|---|---|---|---|---|---|---|---|---|---|---|---|---|
| **356 A** | **356 B** | **358 LA** | **358 LB** | **359 RA** | **360 RA** | **360 RC** | **359 RB** | **359 RC** | **359 RF** | **359 RG** | **359 RH** | **364 A** | **364 B** | | | | |
| **0** | **0** | **0** | **0** | **0** | **0** | **0** | **0** | **0** | **0** | **0** | **0** | **0** | **0** | **0.00** | **0.00** | **14.00** | **0.00** |
| **1** | **0** | **0** | **0** | **4** | **1** | **4** | **2** | **3** | **3** | **0** | **1** | **4** | **2** | **1.79** | **1.58** | **14.00** | **0.44** |
| **1** | **0** | **0** | **0** | **0** | **1** | **1** | **0** | **1** | **1** | **0** | **1** | **1** | **0** | **0.50** | **0.52** | **14.00** | **0.14** |
| **2** | **1** | **0** | **2** | **2** | **2** | **3** | **2** | **2** | **2** | **0** | **2** | **2** | **1** | **1.64** | **0.84** | **14.00** | **0.23** |
| **4** | **1** | **0** | **2** | **6** | **4** | **8** | **4** | **6** | **6** | **0** | **4** | **7** | **3** | | | | |
| | | | | | | | | | | | | | | **3.93** | | | |
| | | | | | | | | | | | | | | **2.53** | | | |
| | | | | | | | | | | | | | | **14** | | | |
| | | | | | | | | | | | | | | **3.61** | | | |
| | | | | | | | | | | | | | | **0.70** | | | |
